# Digital Microsteps as Scalable Adjuncts for Adults Using GLP-1 Receptor Agonists: A Randomized Clinical Trial

**DOI:** 10.1001/jamanetworkopen.2026.0577

**Published:** 2026-03-09

**Authors:** Maya Adam, Louis Fréget, Till Bärnighausen, Fatima Rodriguez, Doron Amsalem, Eleni Linos

**Affiliations:** 1 Department of Pediatrics, Stanford University School of Medicine, Stanford, California; 2 Stanford Center for Digital Health, Department of Medicine, Stanford University, Stanford, California; 3 Heidelberg Institute of Global Health, Medical Faculty and University Hospital, Heidelberg University, Heidelberg, Germany; 4 Université Paris Dauphine-Paris Sciences et Lettres (PSL), Paris, France; 5 Centre pour la recherche économique et ses applications (CEPREMAP), Paris, France; 6 Harvard T.H. Chan School of Public Health, Boston, Massachusetts; 7 Africa Health Research Institute, Somkhele, South Africa; 8 Division of Cardiovascular Medicine, Department of Medicine, Stanford University School of Medicine, Stanford, California; 9 Columbia University Vagelos College of Physicians and Surgeons, Columbia University Irving Medical School, New York, New York; 10 Department of Medicine, Stanford University School of Medicine, Stanford, California; 11 Department of Dermatology, Stanford University School of Medicine, Stanford, California

## Abstract

**Importance:**

With the rapid global uptake of glucagon-like peptide-1 (GLP-1) receptor agonists (GLP-1RAs), scalable behavioral adjuncts are urgently needed to support lifestyle change alongside pharmacotherapy. Microsteps, small behavior change prompts, could be one route to introduce lifestyle change.

**Objective:**

To evaluate whether digitally delivered microsteps, augmented with short video boosters, increases behavioral expectation to adopt lifestyle behaviors among adults using GLP-1 RAs.

**Design, Setting, and Participants:**

This 3-arm online randomized clinical trial was conducted between June 19 and July 1, 2025, with baseline data collection, immediate postexposure, and a 2-week follow-up. Participants were English-speaking adults using GLP-1 RAs in the US, the UK, and other countries and were recruited online through Prolific Academic. Data were collected via the Stanford Medicine Qualtrics platform.

**Interventions:**

Participants received a single exposure to either written microsteps plus a short (approximately 2-minute) storytelling video (arm A) or written microsteps plus a short didactic video (arm B) compared with a do-nothing control condition (arm C). Behavioral nudges focused on small dietary improvements, physical activity, stress management strategies, and sleep hygiene.

**Main Outcomes and Measures:**

The primary outcome was behavioral expectation to adopt health nudges, immediately after exposure and 2 weeks later. The effect on the main outcome of the microsteps intervention vs the control condition was compared; the effect of the intervention with a storytelling vs didactic video was also compared. Secondary outcomes were postexposure hope and happiness, as well as changes (over 2 weeks) in 3 key self-reported diet and exercise behaviors.

**Results:**

Data from 5054 adults using GLP-1 RAs (mean [SD] age, 38.8 [12.6] years; 3361 females [66.5%]) were analyzed. Of these, 3437 (68%) lived in the US and the UK and 1617 (32%) lived in other countries. Immediately after exposure to the interventions, compared with control, standardized effects in SD units across the 8 microsteps expectation outcomes ranged from 0.30 (95% CI, 0.25-0.35) to 0.72 (95% CI, 0.68-0.77) in arm A and from 0.26 (95% CI, 0.21-0.32) to 0.77 (95% CI, 0.72-0.81) in arm B, with smaller effects 2 weeks later. The storytelling video was more effective across 7 of the 8 microsteps (breathe when stressed: arm A, 0.57 [95% CI, 0.52-0.62] vs arm B, 0.50 [95% CI, 0.44-0.55] and go outside for 5 minutes: arm A, 0.53 [95% CI, 0.49-0.58] vs arm B, 0.48 [95% CI, 0.43-0.52]). Immediate increases in hope (arm A, 0.31 [95% CI, 0.27-0.34]; arm B, 0.25 [95% CI, 0.22-0.28]) and happiness (arm A, 0.26 [95% CI, 0.23-0.30]; arm B, 0.22 [95% CI, 0.19-0.25]) were observed but dissipated by 2 weeks. At follow-up, the storytelling video group also reported reduced sugar-sweetened beverage consumption compared with the control group (−0.10 [95% CI, −0.16 to −0.03]).

**Conclusions and Relevance:**

In this randomized clinical trial, a low-cost digital intervention increased expectation to adopt health behaviors among adults using GLP-1 RAs, with effects persisting for 2 weeks. These findings suggest a potential role for the written microsteps intervention plus short video boosters as adjuncts to pharmacotherapy. Longer trials are warranted to determine whether the behavioral expectations stimulated by such interventions may lead to sustained behavior change.

**Trial Registration:**

ClinicalTrials.gov Identifier: NCT06967337

## Key Points

**Question:**

Among adults using glucagon-like peptide-1 receptor agonists (GLP-1 RAs), can a brief digital intervention, consisting of written behavior change prompts accompanied by short videos, boost expectations to adopt health lifestyle behaviors?

**Findings:**

In this randomized clinical trial of 5054 global adults using GLP-1 RAs, single exposure to behavior change prompts with either storytelling or didactic video boosters significantly improved behavioral expectation to adopt health behaviors immediately after exposure, with effects still evident 2 weeks later. The storytelling video showed stronger effects across most domains.

**Meaning:**

The findings of this study demonstrate that even a single, low-cost digital exposure can measurably boost expectations to adopt health lifestyle behaviors in adults using GLP-1 RAs, suggesting that scalable digital microinterventions could complement pharmacotherapy and warrant testing in longer trials.

## Teaser

This randomized clinical trial evaluates whether digital behavior change prompts augmented with short videos increase expectations to adopt lifestyle behaviors in adults using glucagon-like peptide-1 receptor agonists (GLP-1 RAs).

## Introduction

Glucagon-like peptide-1 (GLP-1) receptor agonists (RAs) are increasingly used to treat obesity, type 2 diabetes, and related metabolic disorders.^1^ While GLP-1 medications promote weight loss and improve metabolic health, clinicians fear that the growing reliance on pharmacotherapy may shift attention away from lifestyle modifications that remain critical for protecting long-term cardiometabolic health.^2,3^ Even small behavioral improvements can boost physiologic goals, such as weight management and blood glucose control, in combination with GLP-1 therapy.^3,4,5^

Adults using GLP-1 RAs represent a strategically important subgroup, in which expectations around lifestyle change may be particularly fragile, making them a critical test population for low-burden behavioral adjuncts. Small behavioral nudges may help adults using GLP-1 RAs adopt adjunct lifestyle improvements (including dietary changes, physical activity, stress management, and sleep hygiene) that can synergize with pharmacotherapy.^6,7,8,9,10,11,12^

However, physicians often forgo lifestyle counseling because it requires too much time for minimal perceived gains.^13^ Digitally delivered health microsteps, which are small behavior change prompts designed to be easily adopted, offer a promising, potentially scalable approach.^14,15,16,17,18,19,20^ The approach is informed by the Habit Alteration Model, which is based on 3 key theories of habit formation that help intervention designers understand how users form and break habits.^18^ Yet, to our knowledge, there is limited evidence on brief digital microinterventions for the rapidly growing population of adults using GLP-1 RAs, and we have found no prior studies comparing different short-form video formats as boosters for such prompts.^21^

In early-phase behavioral intervention studies, proximal outcomes such as behavioral expectation (a precondition for behavior change) can be useful for determining whether interventions are likely to justify longer trials with objective outcomes.^19,22,23,24^ Behavioral expectation is considered a superior proximal predictor compared with behavioral intent because it incorporates an individual’s appraisal of the deterrents likely to impede adoption.^19,23,24^ Phrasing questions as: “All things considered, how likely are you to…” allows people to consider the actual barriers that may prevent them from adopting recommended changes.^19,23,24^ Short-term affective and motivational responses to health messages, such as hope and happiness, may also influence message salience and encoding.^25,26,27^

In this study, we tested whether a single exposure to 8 written microsteps, boosted by either a wordless animated storytelling video (arm A) or a short didactic video (arm B), increased behavioral expectation to adopt health microsteps, immediately after exposure (T1) and 2 weeks later (T2), compared with a do-nothing control (arm C). Secondary outcomes were hope and happiness (T1, T2) and changes in 3 self-reported health behaviors from baseline to T2.

## Methods

### Trial Design

The trial protocol ethics approval for this study was obtained from the Stanford University institutional review board. All participants provided electronic informed consent via the Prolific Academic platform prior to participation. This study followed the Consolidated Standards of Reporting Trials (CONSORT) reporting guideline for randomized clinical trials.

This study was a 3-arm, parallel group randomized clinical trial comparing the effect of single exposure to 8 health microsteps, boosted by either a wordless, short animated storytelling video (arm A) or a short didactic video (arm B) vs a do-nothing control arm (arm C) on behavioral expectation to adopt the microsteps. We recruited adult GLP-1 users, in various global regions via an online academic research platform, Prolific Academic, which has large participant pools in the US and the UK and smaller pools in other countries. We collected demographic data and baseline data related to participants’ health behaviors.

Intervention arms A and B both received written messages describing the 8 health microsteps intervention. Arm A also received a 2:21-minute wordless animated storytelling video, whereas arm B received a 2:26-minute didactic video providing information about the intervention. All participants completed 2 visual analog scales measuring hope and happiness, as well as a questionnaire to assess their behavioral expectation of adopting the intervention in the coming 2 weeks (trial protocol in Supplement 2). The microsteps intervention can be found in eFigure 1 in Supplement 1, and the survey instrument is in the eAppendix of Supplement 1.

Control arm C participants completed the demographic and baseline data surveys as well as the behavioral expectation questionnaire and the hope and happiness scales at T1. Two weeks later, participants returned to complete follow-up questionnaires. The trial design is provided in eFigure 2 in Supplement 1.

### Trial Setting and Participant Eligibility Criteria

This study was conducted entirely online. We recruited English-speaking adult using GLP-1 RAs via Prolific Academic from June 19 to July 1, 2025. Participants were paid $4.80. All participants remained anonymous to the investigators (all authors) for the duration of the trial, and each participant was assigned a unique identification number. The written messages, video interventions, and questionnaires were delivered through the Stanford Medicine Qualtrics platform, a survey and data collection platform used for online behavioral research.

### Intervention Description

#### Microsteps

The 8 microsteps were evidence-based behavioral nudges, developed by Thrive Global, a health-promotion platform based in the US.^4,6,7,8,20^ The microsteps (eFigure 1 in Supplement 1) focused on small lifestyle changes related to improved food choices, exercise, sleep, and stress management.

#### Storytelling Booster Video

The storytelling booster video was a short (2 minutes, 21 seconds), 2-dimensional, animated wordless video, created using an approach called short animated storytelling (SAS).^28,29,30^ Because SAS videos are wordless and culturally accessible, they can be useful for overcoming language, literacy, and cultural barriers. Engagement is boosted through the use of classical story structure.^31,32^ The video is intended to complement the written microsteps by enhancing their durability and eliciting positive emotions (hope and happiness). The SAS approach, with key animated scenes mapped along the 3-act storytelling structure, is shown in eFigure 3 in Supplement 1.^33^

#### Didactic Booster Video

The didactic booster video was a 2-minute, 26-second live-action informational video showing excerpts from content created by Thrive Global to support adoption of the microsteps.^34^ The video included a brief rationale for the microsteps, examples of how to integrate movement into daily activities, and a short guided breathing exercise. The narrators of the didactic video were selected for their knowledgeability and trustworthiness, both key components of effective health behavior change messages.^35^

### Explanation for the Choice of Comparators

At T1, we measured immediate intervention effects by comparing outcomes measured after exposure with those measured at baseline, between groups. At T2 (2 weeks), we measured short-term durability using the same approach. Follow-up was intentionally brief because such interventions could be delivered repeatedly at low marginal cost.

### Outcomes and Measurement Tools

The primary outcome was behavioral expectation to adopt each of the 8 recommended microsteps, measured at baseline, immediately after the intervention (T1), and 2 weeks later (T2). Expectation to adopt each of the microsteps was assessed using a 5-point Likert scale, ranging from 1 (extremely unlikely) to 5 (extremely likely). Secondary outcomes included 2 measures of subjective well-being (hope and happiness), scored using a 0-to-100 visual analog scale, an established tool for assessing such constructs, in which higher scores indicate greater feelings of well-being.^36^ We also gathered data at baseline and T2 for 3 self-reported health behaviors: frequency of sugar-sweetened beverage intake, dietary protein intake, and physical activity. These behaviors were measured using a 4-point Likert scale, ranging from 1 (rarely or never) to 4 (daily).

### Sample Size

We calculated the sample size independently for primary and secondary outcomes and selected the most conservative estimate. The microsteps questionnaire included 8 items on a 5-point Likert scale, but with no prior data available, the expected effect size could not be directly estimated. Because our trial tested a single brief exposure, and prior data were limited, we powered the study to detect a very small standardized mean difference (Cohen *d* = 0.10) with a 2-sided α = .05 and 80% power.^37^ The target sample was 6000 participants (2000 per arm), including a priori inflation for attrition.

### Sequence Generation, Allocation Concealment, Blinding, and Implementation

The Stanford Medicine Qualtrics platform used a computer-generated allocation sequence to randomly allocate participants 1:1:1 to the 3 trial arms. All study investigators involved in the data analyses were blinded to the trial arm allocation for the duration of the study.

### Statistical Analysis

Outcomes were standardized (mean [SD], 0 [1]) to express effects in SD units. We estimated intention-to-treat effects using linear regression, with robust SEs, adjusting for baseline values to improve precision. Results are presented as effect estimates with 95% CIs; exact *P* values are reported where relevant.

As a robustness check for the ordinal behavioral outcomes, we also estimated ordered probit models, controlling for baseline values and treatment assignment. (Results are available from the corresponding author.) To address multiplicity, we applied Romano-Wolf stepdown adjusted *P* values separately for the T1 and T2 outcome families (eTables 2 and 3 in Supplement 1). Analyses were conducted in Stata, version 16 (StataCorp LLC), with 2-sided *P* < .05 indicating statistical significance. For descriptive tables only, we summarized the proportion of participants reporting high expectation or frequent engagement using prespecified cut points; for primary analyses, we used the original scales.

#### Subgroup Analyses and Missing Data

Exploratory subgroup analyses compared participants in the US or the UK with other countries using interaction terms between trial arm (A or B) and region (US or UK vs other), controlling for baseline values (eFigures 5 and 6 in Supplement 1). All questions were required for submission, preventing item-level missingness among included participants.

#### Protocol Adherence

Timers on the intervention-delivery platform required that participants remain on the video page for the full video duration before advancing; however, this did not guarantee visual attention or comprehension. Four attention checks were embedded; participants failing more than 1, at either T1 or T2, were excluded. We assessed selective attrition using a probit model of follow-up completion on treatment assignment.

## Results

### Trial Flow

**Figure 1.**
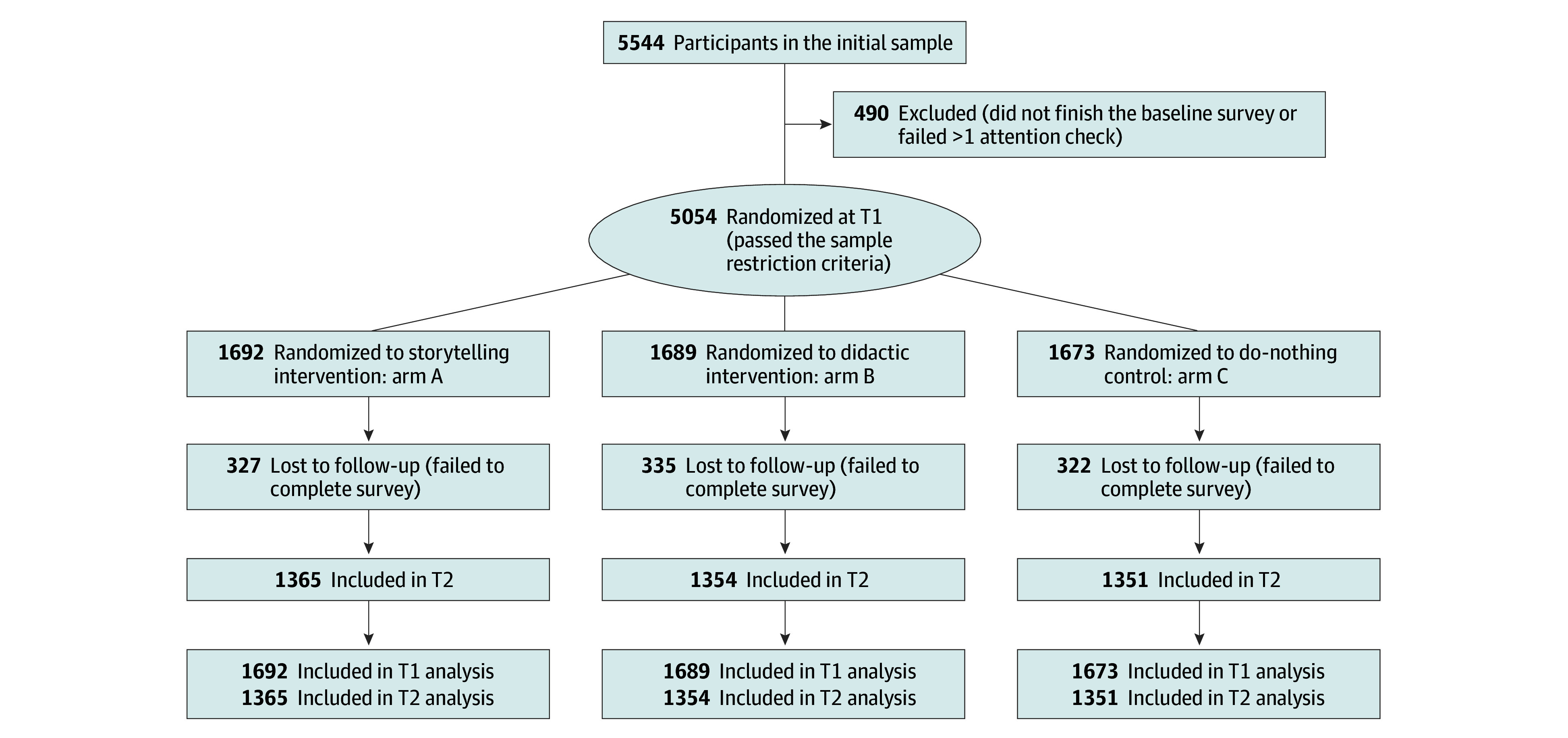
CONSORT Trial Flow Diagram T1 indicates measurement of immediate intervention effects by comparing outcomes after exposure with those at baseline; T2, measurement of short-term durability at 2 weeks after intervention.

We enrolled 5544 adults using GLP-1 RAs between June 19 and July 1, 2025. Of these, 490 (8.8%) did not finish the baseline survey or failed more than 1 attention check. A total of 5054 participants (mean [SD] age, 38.8 [12.6] years; 3361 females [66.5%] and 1693 males [33.5%]) were randomized to the 3 study conditions; 3437 (68%) lived in the US and the UK, and 1617 (32%) lived in other countries. Two weeks later, participants were invited back, via the Prolific Academic platform, to complete the follow-up surveys. In total, 4070 participants (80.5%) returned to complete phase 2. The trial flow is shown in Figure 1. Follow-up completion rates were similar across study arms, with no difference in attrition by assignment.

### Participant Demographics

Across all participants, 3213 (63.6%) had at least some college education. At baseline, 4296 respondents (85.0%) were working part-time or full-time. More than a quarter of participants (1367 [27.0%]) reported difficulty affording basic needs. Baseline characteristics were well-balanced between randomized groups, consistent with successful randomization. Baseline demographic data are shown in the Table, and baseline outcomes data, by study arm, are summarized in eTable 1 in Supplement 1. The distribution of participants by country, with 68% based in the UK and the US and the remaining 32% drawn from other countries around the world, is provided in eFigure 4 in Supplement 1.

**Table.** Descriptive Statistics for Baseline Demographic Variables

Variable	Participants, No. (%)			
	Total sample (N = 5054)	Storytelling: arm A (n = 1692)	Didactic: arm B (n = 1689)	Do-nothing control: arm C (n = 1673)
Age, mean (SD), y	38.8 (12.6)	39.3 (12.8)	38.5 (12.5)	38.7 (12.7)
Sex				
Female	3361 (66.5)	1121 (66.3)	1126 (66.7)	1114 (66.6)
Male	1693 (33.5)	571 (33.7)	563 (33.3)	559 (33.4)
Educational level				
<High school	16 (<1)	8 (<1)	2 (<1)	6 (<1)
High school graduate	347 (6.9)	108 (6.4)	120 (7.1)	119 (7.1)
Some college	631 (12.5)	212 (12.5)	222 (13.1)	197 (11.8)
2-y Degree	321 (6.4)	112 (6.6)	103 (6.1)	106 (6.3)
3-y Degree	398 (7.9)	131 (7.7)	132 (7.8)	135 (8.1)
4-y Degree	1461 (28.9)	479 (28.3)	498 (29.5)	484 (28.9)
Doctorate	402 (8.0)	134 (7.9)	131 (7.8)	137 (8.2)
Professional degree	1478 (29.2)	508 (30.0)	481 (28.5)	489 (29.2)
Employment				
Working full-time	3468 (68.8)	1178 (69.6)	1128 (66.8)	1162 (69.5)
Working part-time	828 (16.4)	284 (16.8)	290 (17.2)	254 (15.2)
Unemployed and looking for work	169 (3.3)	57 (3.4)	64 (3.8)	48 (2.9)
Homemaker or stay-at-home parent	158 (3.1)	39 (2.3)	62 (3.7)	57 (3.4)
Student	141 (2.8)	42 (2.5)	42 (2.5)	57 (3.4)
Retired	182 (3.6)	60 (3.5)	54 (3.2)	68 (4.1)
Other	108 (2.1)	32 (1.9)	49 (2.9)	27 (1.6)
Difficulty affording basic needs	1367 (27.0)	465 (27.5)	462 (27.4)	440 (26.3)

### Effects of the Intervention

At T1, both interventions significantly increased expectation to adopt the microsteps across all domains compared with control (standardized effects in SD units ranged from 0.30 [95% CI, 0.25-0.35] to 0.72 [95% CI, 0.68-0.77] in arm A and from 0.26 [95% CI, 0.21-0.32] to 0.77 [95% CI, 0.72-0.81] in arm B), with larger SD standardized effects observed in the storytelling video group for 7 of 8 of the microsteps. The most pronounced effects were seen for the expectation to set a reminder to go to bed (arm A: 0.72 [95% CI, 0.68-0.77]; arm B: 0.77 [95% CI, 0.72-0.81]) and to reduce caffeine consumption after 2 pm (arm A: 0.59 [95% CI, 0.54-0.63]; arm B: 0.49 [95% CI, 0.45-0.54]). We measured 2 distinct movement-related expectations: scheduling time for movement and adding movement to existing routines. Both showed significant improvements: scheduling time for movement (arm A: 0.54 [95% CI, 0.50-0.59]; arm B: 0.48 [95% CI, 0.43-0.52]) and adding movement to existing routines (arm A: 0.53 [95% CI, 0.49-0.58]; arm B: 0.45 [95% CI, 0.40-0.50]). Effects on hope (arm A: 0.31 [95% CI, 0.27-0.34]; arm B: 0.25 [95% CI, 0.22-0.28]) and happiness (arm A: 0.26 [95% CI, 0.23-0.30]; arm B: 0.22 [95% CI, 0.19-0.25]) were smaller but still statistically significant. These results remained robust after adjusting for multiple hypothesis testing using the Romano-Wolf procedure applied across all T1 outcomes, including both behavioral expectations and emotional states (eTable 2 in Supplement 1).

**Figure 2.**
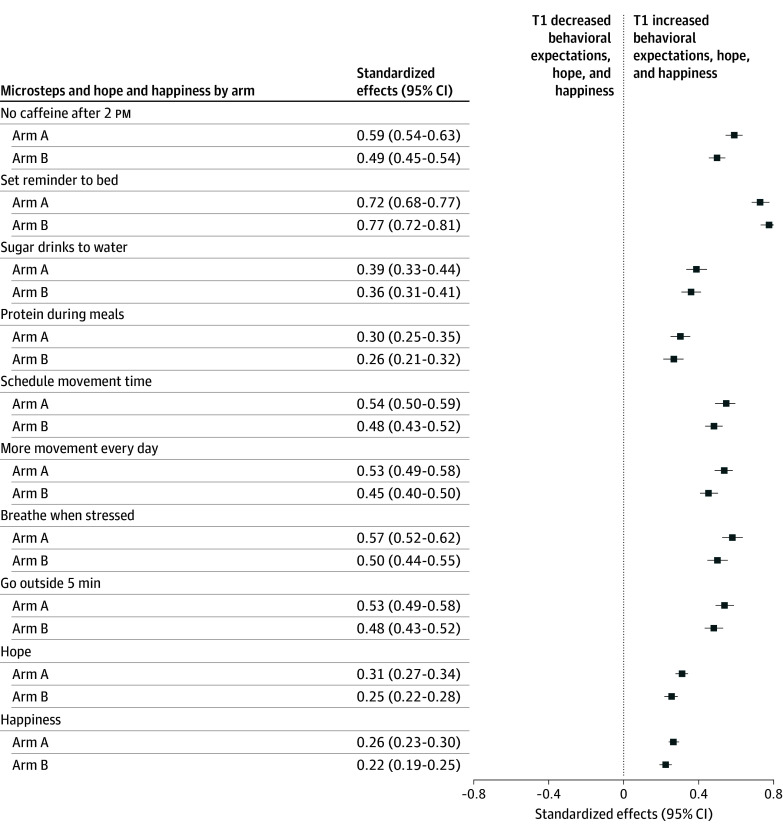
Forest Plot of the Standardized Effects of Microsteps and Video-Based Interventions on Behavioral Expectations to Adopt Them, Hope, and Happiness Immediately After Exposure (T1) All models control for baseline values of each dependent variable (N = 5054 for all regressions). The reference is control message (arm C). Arm A indicates written microsteps and storytelling video booster; arm B, written microsteps and didactic video booster.

Standardized effects of the microsteps and video-based interventions on participants’ behavioral expectations, hope, and happiness, measured immediately after the intervention (T1), relative to the control group, are presented in Figure 2. Direct comparisons between arm A and arm B showed numerically larger effects for the storytelling video across most microsteps (eg, breathe when stressed: arm A, 0.57 [95% CI, 0.52-0.62] vs arm B, 0.50 [95% CI, 0.44-0.55] and go outside for 5 minutes: 0.53 [95% CI, 0.49-0.58] vs arm B, 0.48 [95% CI, 0.43-0.52]), although the trial was not powered for formal noninferiority or superiority testing between active interventions.

**Figure 3.**
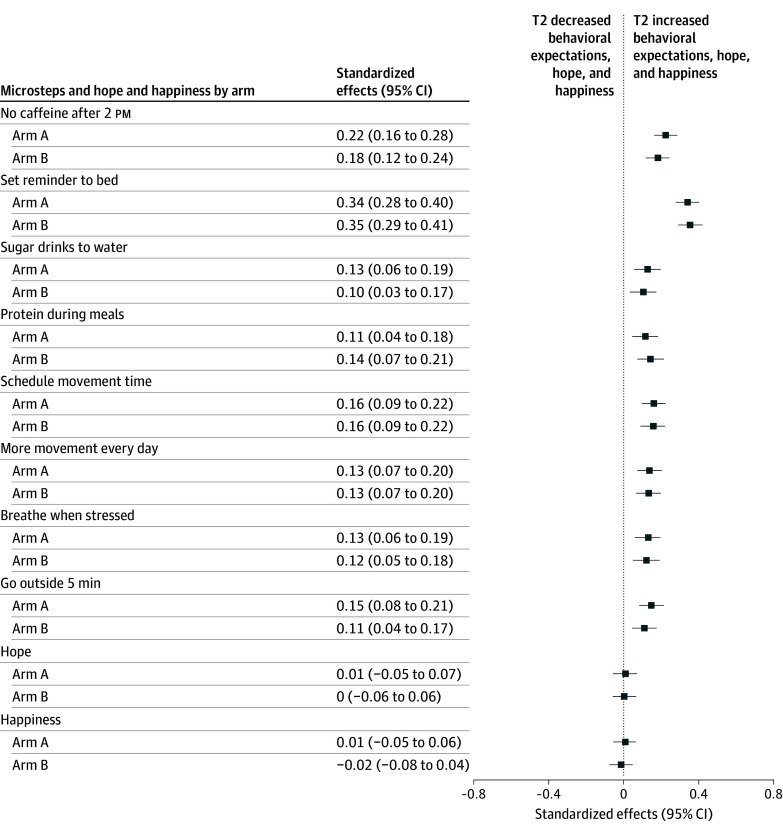
Forest Plot of the Standardized Effects of Microsteps and Video-Based Interventions on Behavioral Expectations to Adopt Them, Hope, and Happiness 2 Weeks After Exposure (T2) All models control for baseline values of each dependent variable (n = 4070 for all regressions). The reference is control message (arm C). Arm A indicates written microsteps and storytelling video booster; arm B, written microsteps and didactic video booster.

Two weeks after exposure to the intervention, at T2, both arms continued to show statistically significant effects on behavioral expectations to adopt microsteps, although effect sizes were attenuated compared with immediate postintervention responses. The mean standardized effect across outcomes declined from 0.45 at T1 to 0.13 at T2, over the 2-week period. At T2, the strongest effects were seen for expectations to set a bedtime reminder (arm A: 0.34 [95% CI, 0.28-0.40]; arm B: 0.35 [95% CI, 0.29-0.41]); reduce caffeine consumption after 2 pm (arm A: 0.22 [95% CI, 0.16-0.28]; arm B: 0.18 [95% CI, 0.12-0.24]); and sustain physical activity, reflected across scheduling time for movement (arm A and arm B: 0.16 [95% CI, 0.09-0.22]), adding movement to daily routines (arm A and arm B: 0.13 [95% CI, 0.07-0.20]), and going outside for at least 5 minutes (arm A: 0.15 [95% CI, 0.08-0.21]; arm B: 0.11 [95% CI, 0.04-0.17]). Effects on hope and happiness were no longer observed at T2. The standardized effects of the written messages plus the 2 video-based interventions on behavioral expectations and secondary outcomes 2 weeks after exposure (T2), relative to the control arm, are shown in Figure 3.

**Figure 4.**
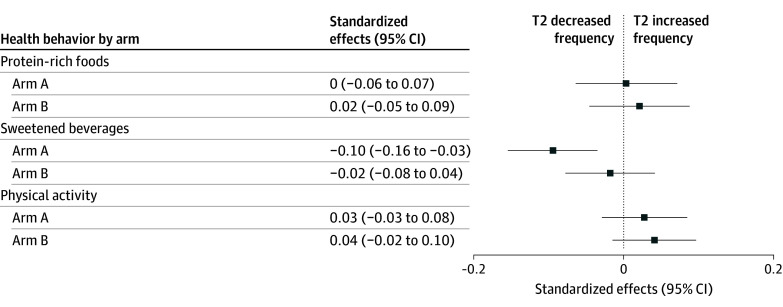
Forest Plot of the Effects of Video-Based Interventions on Self-Reported Health Behaviors 2 Weeks After Intervention (T2) All models control for baseline values of each dependent variable (n = 4070 for all regressions). The reference is control message (arm C). Arm A indicates written microsteps and storytelling video booster; arm B, written microsteps and didactic video booster.

Of the 3 self-reported health behaviors assessed at baseline and T2, 1 showed a statistically significant effect; participants in the storytelling video group (arm A) reported reduced frequency of consuming sugar-sweetened beverages compared with the control group (−0.10 [95% CI, −0.16 to −0.03]). There were no statistically significant effects on physical activity and dietary protein intake. Importantly, the effect on sugar-sweetened beverage consumption remained robust after adjusting for multiple hypotheses testing using the Romano-Wolf stepdown procedure applied to the full set of T2 outcomes (eTable 3 in Supplement 1). The effects of the written messages plus the 2 video-based interventions on the 3 self-reported health behaviors measured 2 weeks after exposure (T2) are shown in Figure 4.

### Cost and Subgroup Analyses

Both booster videos were produced for less than $3500 each. Effects on expectations were observed in both the US or the UK and other countries, with broadly similar patterns (eFigure 5 in Supplement 1). Exploratory analyses suggested larger reductions in sugar-sweetened beverage consumption outside of the US or the UK for arm A; subgroup estimates are provided in eFigures 5 and 6 in Supplement 1.

## Discussion

In this fully online randomized clinical trial of 5054 adults using GLP-1 RAs recruited in June to July 2025, a single exposure to written microsteps, boosted by a short video, increased behavioral expectation to adopt recommended lifestyle nudges immediately after exposure, with attenuated but detectable effects at 2 weeks. Across most domains, the storytelling video booster yielded slightly larger effects than the didactic booster.

The findings of this study are particularly encouraging given the light-touch nature of the intervention. Participants received a single, short exposure (a microdose of behavioral guidance), yet the intervention generated immediate effects on a proximal indicator of behavior change, with durability after 2 weeks. Given global trends in digital adoption, this suggests a promising role for digital health microinterventions. With measurable effects emerging from a single exposure, it is possible that more sustained or reinforced versions could yield larger and even more durable impacts. Future long-term studies are needed to evaluate sustained behavior change and clinical outcomes.

We also observed small but statistically significant improvements in the exploratory secondary outcomes of hope and happiness at T1, which may be relevant, as affective engagement can support encoding and short-term retention of health messages.^38^ However, these findings should be interpreted cautiously, given the self-report context and the potential for demand characteristics.

### Limitations

This trial has important limitations. First, outcomes were proximal indicators of behavior change and self-reported behaviors, both of which could be influenced by demand characteristics in an unblinded, compensated survey context. Second, the microsteps and questionnaires were English language, limiting generalizability to non–English-speaking adults using GLP-1 RAs. Third, the do-nothing control may not reflect usual care and may overestimate incremental benefit relative to settings in which lifestyle guidance is routinely provided. Fourth, while nonskippable playback timers were used to ensure complete playing of both videos, we could not verify that participants actually watched during video playback. Also, participants were recruited online and may not represent all adults using GLP-1 RAs, particularly those with limited digital access. As a digitally delivered intervention, it may be less accessible to individuals with limited internet access or low digital engagement, indicating the need for complementary nondigital strategies to support all individuals using GLP-1 RAs. Additionally, while Prolific Academic offered us access to a large, international adult test population, with minimal loss to follow-up, the platform draws mostly from large participant pools in the US and the UK, which limited our ability to recruit equal numbers of participants across all global regions.

## Conclusions

In this randomized clinical trial of adults using GLP-1 RAs, a brief digital microsteps intervention paired with short video boosters increased behavioral expectation to adopt adjunct lifestyle behaviors, with effects persisting for 2 weeks, although attenuated. These findings support evaluation of repeated or adaptive dosing strategies and longer trials incorporating objective behavioral outcomes. As the use of GLP-1 medications increases globally, developing scalable behavior change interventions is a growing public health priority. Stepwise, cost-effective approaches, such as a scalable digital microsteps intervention and short-form videos, could support GLP-1 therapy regimens and contribute to existing efforts aimed at addressing the global burden of noncommunicable disease.

## Notes

Supplement 1. **eFigure 1.** Microsteps **eFigure 2. **Trial Design **eFigure 3.** Key Scenes From the Storytelling Booster Video (Intervention Arm A), Shown Beside Their Corresponding Story Beats in Classical 3-Act Storytelling Structure **eFigure 4.** Global Distribution of the Participants **eFigure 5. **Standardized Effects of the Microsteps and Video-Based Interventions on Behavioral Expectations, Hope, and Happiness Immediately After Exposure (T1), by Geographic Origin of the Respondents: US or UK or Rest of the World **eFigure 6.** Standardized Effects of the Microsteps and Video-Based Interventions on Self-Reported Health Behaviors at T2, by Geographic Origin of the Respondents: US or UK or Rest of the World **eTable 1. **Descriptive Statistics for Baseline Outcome Variables **eTable 2.** Multiple Testing *P* Value Adjustment for T1 Outcomes (Romano Wolf Method) **eTable 3.** Multiple Testing *P* Value Adjustment for T2 Outcomes (Romano Wolf Method) **eAppendix.** GLP Microsteps (Baseline Survey)

Supplement 2.
Trial Protocol

Supplement 3.
Data Sharing Statement
