# Supplementary material for: Digital Microsteps as Scalable Adjuncts for Adults Using GLP-1 Receptor Agonists: A Randomized Clinical Trial
Source: JAMA Netw Open. 2026 Mar 9;9(3):e260577. doi: 10.1001/jamanetworkopen.2026.0577 (PMC12973101; doi:10.1001/jamanetworkopen.2026.0577)
Supplement: Supplement 1. — eFigure 1. Microsteps eFigure 2. Trial Design eFigure 3. Key Scenes From the Storytelling Booster Video (Intervention Arm A), Shown Beside Their Corresponding Story Beats in Classical 3-Act Storytelling Structure eFigure 4. Global Distribution of the Participants eFigure 5. Standardized Effects of the Microsteps and Video-Based Interventions on Behavioral Expectations, Hope, and Happiness Immediately After Exposure (T1), by Geographic Origin of the Respondents: US or UK or Rest of the World eFigure 6. Standardized Effects of the Microsteps and Video-Based Interventions on Self-Reported Health Behaviors at T2, by Geographic Origin of the Respondents: US or UK or Rest of the World eTable 1. Descriptive Statistics for Baseline Outcome Variables eTable 2. Multiple Testing P Value Adjustment for T1 Outcomes (Romano Wolf Method) eTable 3. Multiple Testing P Value Adjustment for T2 Outcomes (Romano Wolf Method) eAppendix. GLP Microsteps (Baseline Survey) [file jamanetwopen-e260577-s001.pdf]

Supplemental Online Content

Adam M, Fréget L, Bärnighausen T, Rodriguez F, Amsalem D, Linos E. Digital Microsteps as scalable adjuncts for adults using GLP-1 receptor agonists: a randomized clinical trial. *JAMA Netw Open*. 2026;9(3):e260577. doi:10.1001/jamanetworkopen.2026.0577

eFigure 1. Microsteps
eFigure 2. Trial Design
eFigure 3. Key Scenes From the Storytelling Booster Video (Intervention Arm A), Shown Beside Their Corresponding Story Beats in Classical 3-Act Storytelling Structure
eFigure 4. Global Distribution of the Participants
eFigure 5. Standardized Effects of the Microsteps and Video-Based Interventions on Behavioral Expectations, Hope, and Happiness Immediately After Exposure (T1), by Geographic Origin of the Respondents: US or UK or Rest of the World
eFigure 6. Standardized Effects of the Microsteps and Video-Based Interventions on Self-Reported Health Behaviors at T2, by Geographic Origin of the Respondents: US or UK or Rest of the World
eTable 1. Descriptive Statistics for Baseline Outcome Variables
eTable 2. Multiple Testing *P* Value Adjustment for T1 Outcomes (Romano Wolf Method)
eTable 3. Multiple Testing *P* Value Adjustment for T2 Outcomes (Romano Wolf Method)
eAppendix. GLP Microsteps (Baseline Survey)

This supplemental material has been provided by the authors to give readers additional information about their work.

© 2026 Adam M et al. *JAMA Network Open*.

eFigure 1. Microsteps

For better sleep:
Avoid caffeine after 2 p.m.
Caffeine can stay in your system for hours, making it harder to fall asleep.

Set an alarm for 30 minutes before your bedtime. When you think of sleep as an actual appointment, you're much more likely to grant it the time it deserves. Setting an alarm reminds you that if you're going to get to bed on time, you need to start wrapping things up.

For better nutrition:
Swap sugary drinks for water (can be infused with lemon, mint, or berries).
This cuts excess sugar while keeping hydration enjoyable and refreshing.

Make sure you have a source of protein during your daytime meals.
Eating protein such as lean chicken, salmon, eggs or edamame helps maintain muscle mass, support metabolism, and stabilize energy throughout the day.

For more movement:
Schedule time on your calendar for movement. You wouldn't miss an important meeting or doctor's appointment, so blocking out movement time on your calendar in the same way can help you shift your mindset to prioritize your physical well-being.

Add movement to everyday activities.
Stacking physical activity on top of everyday tasks (like walking while taking a call or doing squats while brushing your teeth) is a great way to add movement with no extra time.

To help manage stressful moments:
Recognize when you're feeling stressed. Then pause, take a deep breath and let it out slowly. Just a few minutes of deep, mindful breathing can lower blood pressure and help us react to stressful situations more effectively.

Go outside for 5 minutes — and leave your devices inside.
Simply being outside can boost your mood and reduce stress. Plus, disconnecting from your devices can give you a moment to reflect and decompress.

© 2026 Adam M et al. *JAMA Network Open*.

GLP Microsteps (Baseline Survey)

Start of Block: Introduction

GLP_type Thank you for agreeing to take part in this survey. Which GLP-1 Agonist(s) do you have experience taking?

- ○ Ozempic – semaglutide (4)
- ○ Wegovy – semaglutide (5)
- ○ Mounjaro – tirzepatide (6)
- ○ Zepbound – tirzepatide (7)
- ○ Adlyxin – lixisenatide (8)
- ○ Rybelsus – semaglutide (9)
- ○ Trulicity – dulaglutide (10)
- ○ Saxenda – liraglutide (11)
- ○ Victoza – liraglutide (12)
- ○ Bydureon – exenatide (13)
- ○ Byetta – exenatide (14)
- ○ Other (15)

End of Block: Introduction

Start of Block: GLP_Beliefs and Experiences

Instructions Please choose the answer that best matches your thoughts and experiences with regard to GLP-1 Agonists.

© 2026 Adam M et al. *JAMA Network Open*.

Stanford MEDICINE | Qualtrics

Weight Taking a GLP-1 Agonist helped me lose weight.

- ◯ Strongly disagree (1)
- ◯ Somewhat disagree (2)
- ◯ Neither agree nor disagree (3)
- ◯ Somewhat agree (4)
- ◯ Strongly agree (5)

Beliefs_1 I believe GLP-1 medications reduce the risk of heart disease or stroke.

- ◯ Strongly disagree (1)
- ◯ Somewhat disagree (6)
- ◯ Neither agree nor disagree (3)
- ◯ Somewhat agree (4)
- ◯ Strongly agree (5)

Beliefs_2 I feel that GLP-1 medications are more effective than lifestyle changes alone.

- Strongly disagree (1)
- Somewhat disagree (6)
- Neither agree nor disagree (3)
- Somewhat agree (4)
- Strongly agree (5)

Beliefs_3 I trust the long-term safety of GLP-1 medications.

- Strongly disagree (1)
- Somewhat disagree (6)
- Neither agree nor disagree (3)
- Somewhat agree (4)
- Strongly agree (5)

Experiences_1 I have considered stopping GLP-1 therapy due to cost concerns.

- Strongly disagree (1)
- Somewhat disagree (6)
- Neither agree nor disagree (3)
- Somewhat agree (4)
- Strongly agree (5)

© 2026 Adam M et al. *JAMA Network Open*.

Experiences_2 I have/had regular follow-ups to monitor my response to GLP-1 therapy.

- Strongly disagree (1)
- Somewhat disagree (6)
- Neither agree nor disagree (3)
- Somewhat agree (4)
- Strongly agree (5)

Experiences_3 I feel there is social stigma associated with taking weight-loss medications.

- Strongly disagree (1)
- Somewhat disagree (6)
- Neither agree nor disagree (3)
- Somewhat agree (4)
- Strongly agree (5)

© 2026 Adam M et al. *JAMA Network Open*.

Experiences_4 I am likely to continue GLP-1 therapy long-term if recommended.

- Strongly disagree (1)
- Somewhat disagree (6)
- Neither agree nor disagree (3)
- Somewhat agree (4)
- Strongly agree (5)

Experiences_5 I wish I had more support taking my GLP-1 medication (like a digital coach or other support program)

- Strongly disagree (1)
- Somewhat disagree (6)
- Neither agree nor disagree (3)
- Somewhat agree (4)
- Strongly agree (5)

© 2026 Adam M et al. *JAMA Network Open*.

Experiences_6 Taking a GLP-1 Agonist helped me manage my blood sugar.

- Strongly disagree (1)
- Somewhat disagree (6)
- I don't know (3)
- Somewhat agree (4)
- Strongly agree (5)

Support_1 I use a mobile app or wearables to track my progress while on GLP-1 medications.

- Yes (1)
- No (7)

Support_2 I would prefer a telehealth appointment over an in-person visit to initiate GLP-1 therapy.

- Yes (1)
- No (7)

Support_3 I would prefer a telehealth appointment over an in-person visit for follow-up for GLP-1 care.

- Yes (1)
- No (7)

End of Block: GLP_Beliefs and Experiences

© 2026 Adam M et al. *JAMA Network Open*.

Stanford MEDICINE | Qualtrics

Start of Block: Demographics

Year of birth What year were you born?

▼ 1925 (6) ... 2007 (88)

Country_Res Which country do you live in?

- ◯ USA (1)
- ◯ UK (2)
- ◯ Other (3) ____

Gender What is your gender?

- ◯ Male (1)
- ◯ Female (2)
- ◯ Other (3) ____

© 2026 Adam M et al. *JAMA Network Open*.

Education What is your highest level of education?

- Less than High School (1)
- High school graduate (2)
- Some college (3)
- 2 year degree (4)
- 3 year degree (8)
- 4 year degree (5)
- Professional degree (6)
- Doctorate (7)

Employment What best describes your employment status over the last three months?

- Working full-time (1)
- Working part-time (2)
- Unemployed and looking for work (3)
- A homemaker or stay-at-home parent (4)
- Student (5)
- Retired (6)
- Other (7)

Income How difficult is it for you to afford your basic needs?

- Strongly disagree (6)
- Somewhat disagree (7)
- Neither agree nor disagree (8)
- Somewhat agree (9)
- Strongly agree (10)

End of Block: Demographics

Start of Block: BaseLn_Behaviors

BaseLn_Sweet_Drink How often do you choose to drink sweetened beverages (such as soda, energy drinks, or sweetened tea/coffee) in a typical day?

- Rarely or never (1)
- Occasionally (2)
- Several times a week (3)
- Daily (4)

BaseLn_Protein How often do you include protein-rich foods (such as meat, dairy, legumes, or plant-based alternatives) in each of your meals?

- Rarely or never (1)
- Occasionally (2)
- Several times a week (3)
- Daily (4)

BaseLn_Movemnt_Activ How often do you engage in physical activity that gets your heart rate up (such as walking briskly, jogging, cycling, or strength training)?

- ◯ Rarely or never (1)
- ◯ Occasionally (2)
- ◯ Several times a week (3)
- ◯ Daily (4)

End of Block: BaseLn_Behaviors

Start of Block: BaseLn_Thermometers

BaseLn_Hope Using a scale from zero to 100, please tell us how hopeful you feel at this moment in time. As you do this task, think of an imaginary thermometer. The more hopeful you feel, the higher the number you should give it. The less hopeful you feel, the lower the number. If you feel neither hopeful nor hopeless, rate it 50.

	0 10 20 30 40 50 60 70 80 90 100
How hopeful do you feel? ()	

BaseLn_Happiness Using a scale from zero to 100, please tell us about how happy you feel at this moment. As you do this task, think of an imaginary thermometer. The happier you feel, the higher the number you should give it. The less happy you feel, the lower the number. If you feel neither happy nor unhappy, rate it 50.

	0 10 20 30 40 50 60 70 80 90 100
How happy do you feel? ()	

End of Block: BaseLn_Thermometers

© 2026 Adam M et al. *JAMA Network Open*.

Stanford MEDICINE | Qualtrics

Start of Block: BaseLn_Health Behaviors

Intro In the coming weeks, all things considered, how likely is it that you will do the following things?

BaseLn_Caffeine(R) Drink Caffeine after 2pm.

- ○ Extremely unlikely (1)
- ○ Somewhat unlikely (2)
- ○ Neither likely nor unlikely (3)
- ○ Somewhat likely (4)
- ○ Extremely likely (5)

BaseLn_BedAlarm Set an alarm that reminds me to go to bed.

- ○ Extremely unlikely (1)
- ○ Somewhat unlikely (2)
- ○ Neither likely nor unlikely (3)
- ○ Somewhat likely (4)
- ○ Extremely likely (5)

© 2026 Adam M et al. *JAMA Network Open*.

BaseLn_SugarDrinks Swap sugary drinks for water (can be infused with lemon, mint, or berries).

- Extremely unlikely (1)
- Somewhat unlikely (2)
- Neither likely nor unlikely (3)
- Somewhat likely (4)
- Extremely likely (5)

BaseLn_Protein Make sure I have a source of protein during my daytime meals.

- Extremely unlikely (1)
- Somewhat unlikely (2)
- Neither likely nor unlikely (3)
- Somewhat likely (4)
- Extremely likely (5)

© 2026 Adam M et al. *JAMA Network Open*.

BaseLn_Schedule_move Schedule time on my calendar for movement.

- Extremely unlikely (1)
- Somewhat unlikely (2)
- Neither likely nor unlikely (3)
- Somewhat likely (4)
- Extremely likely (5)

BaseLn_Adding_movmnt Consciously *add* movement to an everyday activity (like walking while I take a work call or doing squats while brushing my teeth).

- Extremely unlikely (1)
- Somewhat unlikely (2)
- Neither likely nor unlikely (3)
- Somewhat likely (4)
- Extremely likely (5)

© 2026 Adam M et al. *JAMA Network Open*.

Stanford MEDICINE | Qualtrics

BaseLn_Breathe Recognize when I'm feeling stressed, pause, take a deep breath and let it out slowly.

- Extremely unlikely (1)
- Somewhat unlikely (2)
- Neither likely nor unlikely (3)
- Somewhat likely (4)
- Extremely likely (5)

Attn_1 This is an attention check. Please select "Extremely likely".

- Extremely unlikely (1)
- Somewhat unlikely (2)
- Neither likely nor unlikely (3)
- Somewhat likely (4)
- Extremely likely (5)

BaseLn_5minutes Go outside for 5 minutes and leave my devices inside.

- Extremely unlikely (1)
- Somewhat unlikely (2)
- Neither likely nor unlikely (3)
- Somewhat likely (4)
- Extremely likely (5)

End of Block: BaseLn_Health Behaviors

Start of Block: Int_A Microsteps+VideoThr

Time_tracker_A Timing
First Click (1)
Last Click (2)
Page Submit (3)
Click Count (4)

Thrive Video Please make sure your **volume is turned up** and watch the video all the way through--

Page Break

Microsteps_written Here are some **proven "Microsteps"** that you can choose to adopt. Studies show they small changes have helped many people to lead **happier, healthier lives.** **For better sleep**: **Avoid caffeine after 2 p.m.** Caffeine can stay in your system for hours, making it harder to fall asleep. **Set an alarm for 30 minutes before your bedtime.** When you think of sleep as an actual appointment, you're much more likely to grant it the time it deserves. Setting an alarm reminds you that if you're going to get to bed on time, you need to start wrapping things up. **For better nutrition: Swap sugary drinks for water (can be infused with lemon, mint, or berries).** This cuts excess sugar while keeping hydration enjoyable and refreshing. **Make sure you have a source of protein during your daytime meals.** Eating protein such as lean chicken, salmon, eggs or edamame helps maintain muscle mass, support metabolism, and stabilize energy throughout the day. **For more movement: Schedule time on your calendar for movement.** You wouldn't miss an important meeting or doctor's appointment, so blocking out movement time on your calendar in the same way can help you shift your mindset to prioritize your physical well-being. **Add movement to everyday activities.** Stacking physical activity on top of everyday tasks (like walking while taking a call or doing squats while brushing your teeth) is a great way to add movement with no extra time. **To help manage stressful moments: Recognize when you're feeling stressed. Then pause, take a deep breath and let it out slowly.** Just a few minutes of deep, mindful breathing can lower blood pressure and help us react to stressful situations more effectively. **Go outside for 5 minutes — and leave your devices inside.** Simply being outside can boost your mood and reduce stress. Plus, disconnecting from your devices can give you a moment to reflect and decompress.

End of Block: Int_A Microsteps+VideoThr

Start of Block: Int_B Microsteps+Video

TimeTrackerB Timing
First Click (1)
Last Click (2)
Page Submit (3)
Click Count (4)

SAS Video
Please make sure your **volume is turned up** and watch the video all the way through. You will be asked some additional questions after watching the video. Thanks for sticking with us! We really appreciate your time.

Page Break

© 2026 Adam M et al. *JAMA Network Open*.

Miscrosteps_written Here are some **proven "Microsteps"** that you can choose to adopt. Studies show they small changes have helped many people to lead **happier, healthier lives.** **For better sleep**: **Avoid caffeine after 2 p.m.** Caffeine can stay in your system for hours, making it harder to fall asleep. **Set an alarm for 30 minutes before your bedtime.** When you think of sleep as an actual appointment, you're much more likely to grant it the time it deserves. Setting an alarm reminds you that if you're going to get to bed on time, you need to start wrapping things up. **For better nutrition: Swap sugary drinks for water (can be infused with lemon, mint, or berries).** This cuts excess sugar while keeping hydration enjoyable and refreshing. **Make sure you have a source of protein during your daytime meals.** Eating protein such as lean chicken, salmon, eggs or edamame helps maintain muscle mass, support metabolism, and stabilize energy throughout the day. **For more movement: Schedule time on your calendar for movement.** You wouldn't miss an important meeting or doctor's appointment, so blocking out movement time on your calendar in the same way can help you shift your mindset to prioritize your physical well-being. **Add movement to everyday activities.** Stacking physical activity on top of everyday tasks (like walking while taking a call or doing squats while brushing your teeth) is a great way to add movement with no extra time. **To help manage stressful moments: Recognize when you're feeling stressed. Then pause, take a deep breath and let it out slowly.** Just a few minutes of deep, mindful breathing can lower blood pressure and help us react to stressful situations more effectively. **Go outside for 5 minutes — and leave your devices inside.** Simply being outside can boost your mood and reduce stress. Plus, disconnecting from your devices can give you a moment to reflect and decompress.

End of Block: Int_B Microsteps+Video

Start of Block: T1 Control Message

T1CntrlMessage Thank you. For **quality control purposes**, we would appreciate it if you would fill out the questions below one more time. After doing so, you'll be done for today! Thanks again.

End of Block: T1 Control Message

Start of Block: T1_Thermometers

T1_Hope Using a scale from zero to 100, please tell us how hopeful you feel at this moment in time. As you do this task, think of an imaginary thermometer. The more hopeful you feel, the higher the number you should give it. The less hopeful you feel, the lower the number. If you feel neither hopeful nor hopeless, rate it 50.

0 10 20 30 40 50 60 70 80 90 100

How hopeful do you feel? ()	

T1_Happiness Using a scale from zero to 100, please tell us about how happy you feel at this moment. As you do this task, think of an imaginary thermometer. The happier you feel, the higher the number you should give it. The less happy you feel, the lower the number. If you feel neither happy nor unhappy, rate it 50.

	0 10 20 30 40 50 60 70 80 90 100
How happy do you feel? ()	

End of Block: T1_Thermometers

Start of Block: T1_Health Behaviors

Intro In the coming weeks, all things considered, how likely is it that you will do the following things?

T1_Caffeine(R) Drink Caffeine after 2pm.

- Extremely unlikely (1)
- Somewhat unlikely (2)
- Neither likely nor unlikely (3)
- Somewhat likely (4)
- Extremely likely (5)

Stanford MEDICINE | Qualtrics

T1_BedAlarm Set an alarm that reminds me to go to bed.

- Extremely unlikely (1)
- Somewhat unlikely (2)
- Neither likely nor unlikely (3)
- Somewhat likely (4)
- Extremely likely (5)

T1_SugarDrinks Swap sugary drinks for water (can be infused with lemon, mint, or berries).

- Extremely unlikely (1)
- Somewhat unlikely (2)
- Neither likely nor unlikely (3)
- Somewhat likely (4)
- Extremely likely (5)

Attn_2 This is an attention check. Please select "Extremely unlikely".

- Extremely unlikely (1)
- Somewhat unlikely (2)
- Neither likely nor unlikely (3)
- Somewhat likely (4)
- Extremely likely (5)

© 2026 Adam M et al. *JAMA Network Open*.

T1_Protein Make sure I have a source of protein during my daytime meals.

- Extremely unlikely (1)
- Somewhat unlikely (2)
- Neither likely nor unlikely (3)
- Somewhat likely (4)
- Extremely likely (5)

T1_Schedule_move Schedule time on my calendar for movement.

- Extremely unlikely (1)
- Somewhat unlikely (2)
- Neither likely nor unlikely (3)
- Somewhat likely (4)
- Extremely likely (5)

T1_Adding_movmnt Consciously *add* movement to an everyday activity (like walking while I take a work call or doing squats while brushing my teeth).

- Extremely unlikely (1)
- Somewhat unlikely (2)
- Neither likely nor unlikely (3)
- Somewhat likely (4)
- Extremely likely (5)

T1_Breathe Recognize when I'm feeling stressed, pause, take a deep breath and let it out slowly.

- Extremely unlikely (1)
- Somewhat unlikely (2)
- Neither likely nor unlikely (3)
- Somewhat likely (4)
- Extremely likely (5)

© 2026 Adam M et al. *JAMA Network Open*.

T1_5min Go outside for 5 minutes and leave my devices inside.

- ◯ Extremely unlikely (1)
- ◯ Somewhat unlikely (2)
- ◯ Neither likely nor unlikely (3)
- ◯ Somewhat likely (4)
- ◯ Extremely likely (5)

End of Block: T1_Health Behaviors

---

eFigure 2. Trial Design

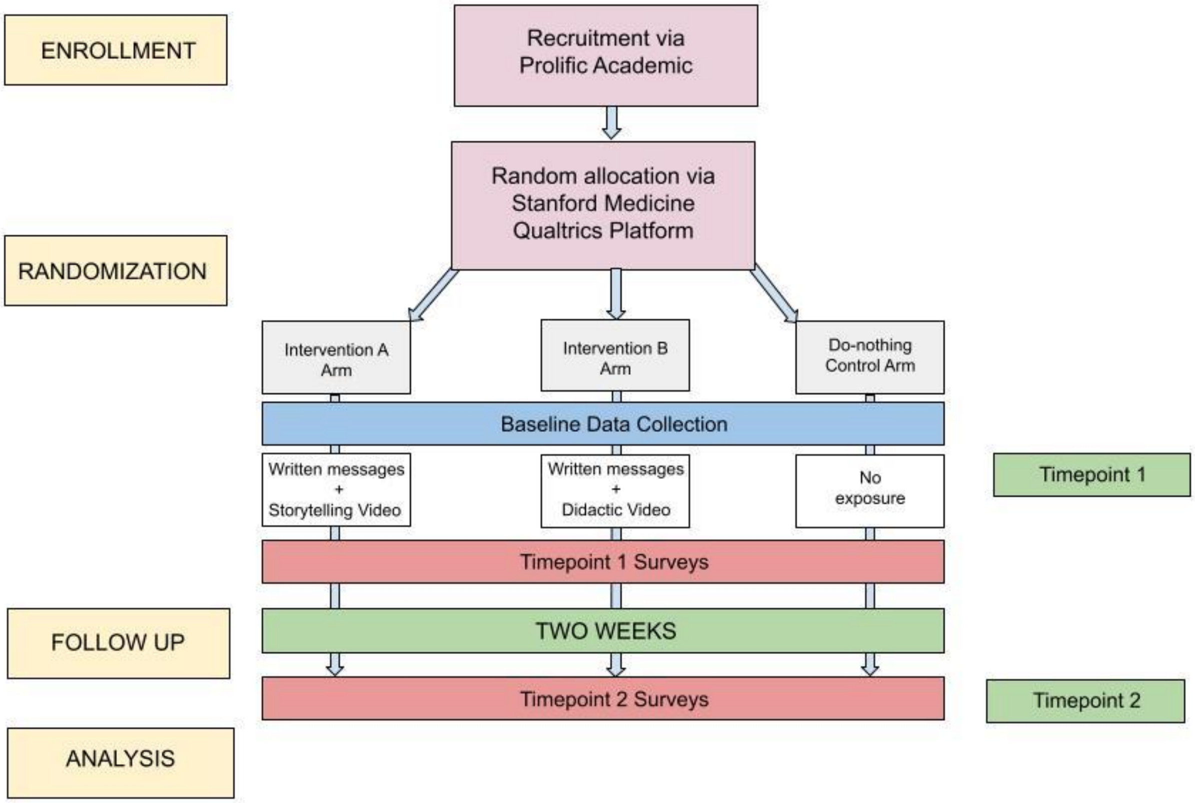

© 2026 Adam M et al. *JAMA Network Open*.

eFigure 3. Key Scenes From the Storytelling Booster Video (Intervention Arm A), Shown Beside Their Corresponding Story Beats in Classical 3-Act Storytelling Structure

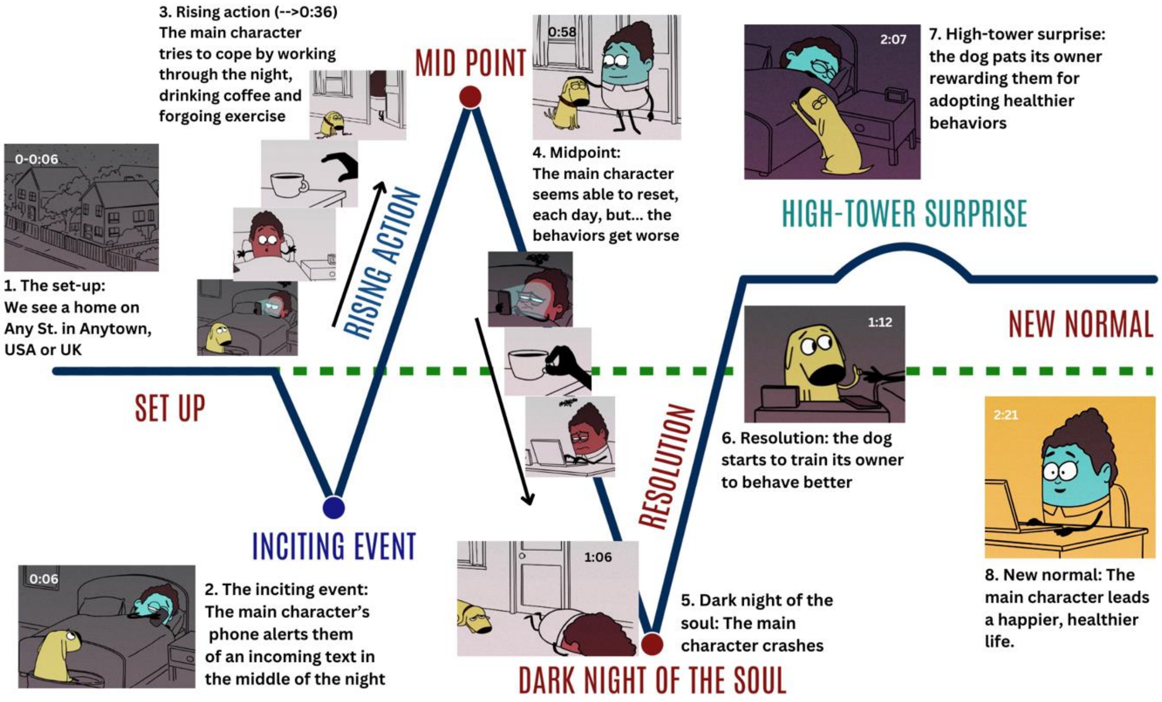

© 2026 Adam M et al. *JAMA Network Open*.

eFigure 4. Global Distribution of the Participants

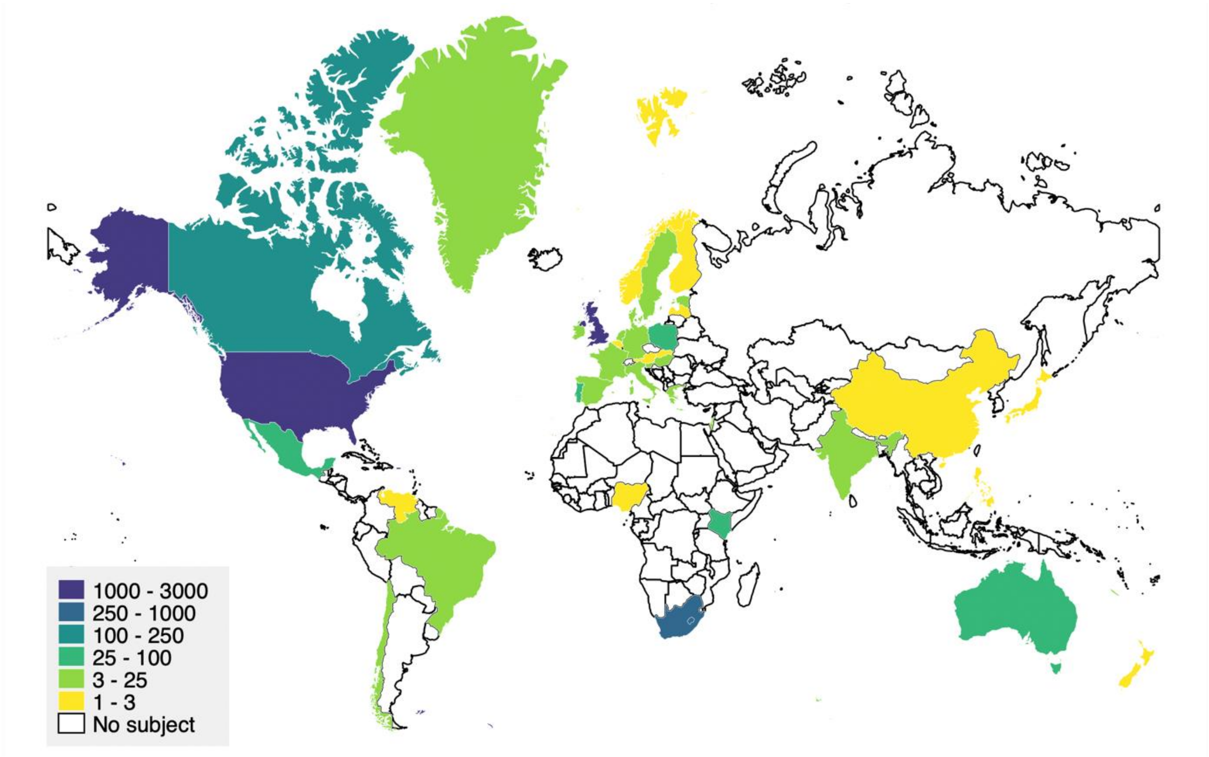

© 2026 Adam M et al. *JAMA Network Open*.

eFigure 5. Standardized Effects of the Microsteps and Video-Based Interventions on Behavioral Expectations, Hope, and Happiness Immediately After Exposure (T1), by Geographic Origin of the Respondents: US or UK (Above) or Rest of the World (Below)

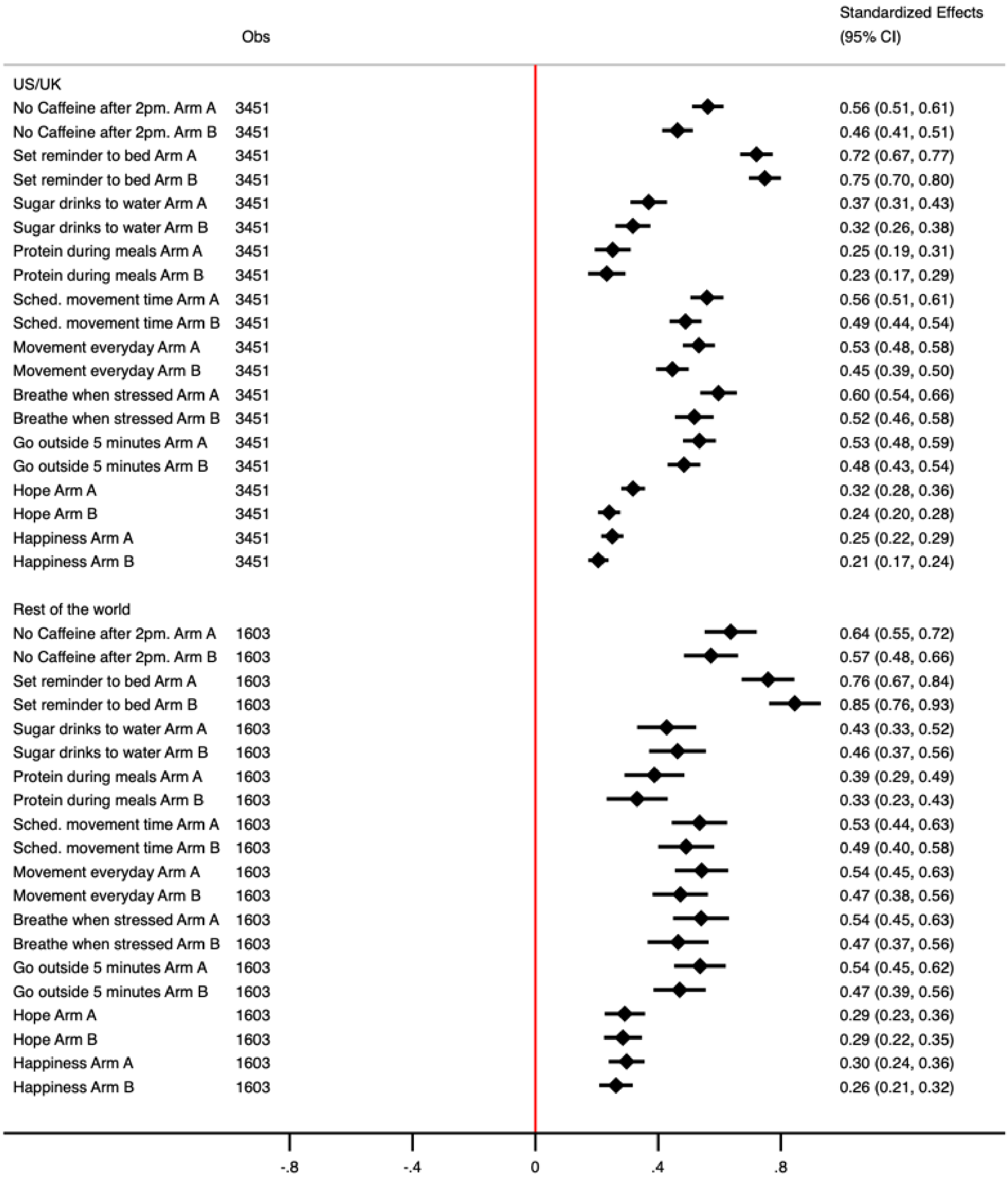

Note: Arm A refers to Written Microsteps + storytelling video booster and Arm B to Written Microsteps + didactic video booster.

© 2026 Adam M et al. *JAMA Network Open*.

eFigure 6. Standardized Effects of the Microsteps and Video-Based Interventions on Self-Reported Health Behaviors at T2, by Geographic Origin of the Respondents: US or UK (Above) or Rest of the World (Below)

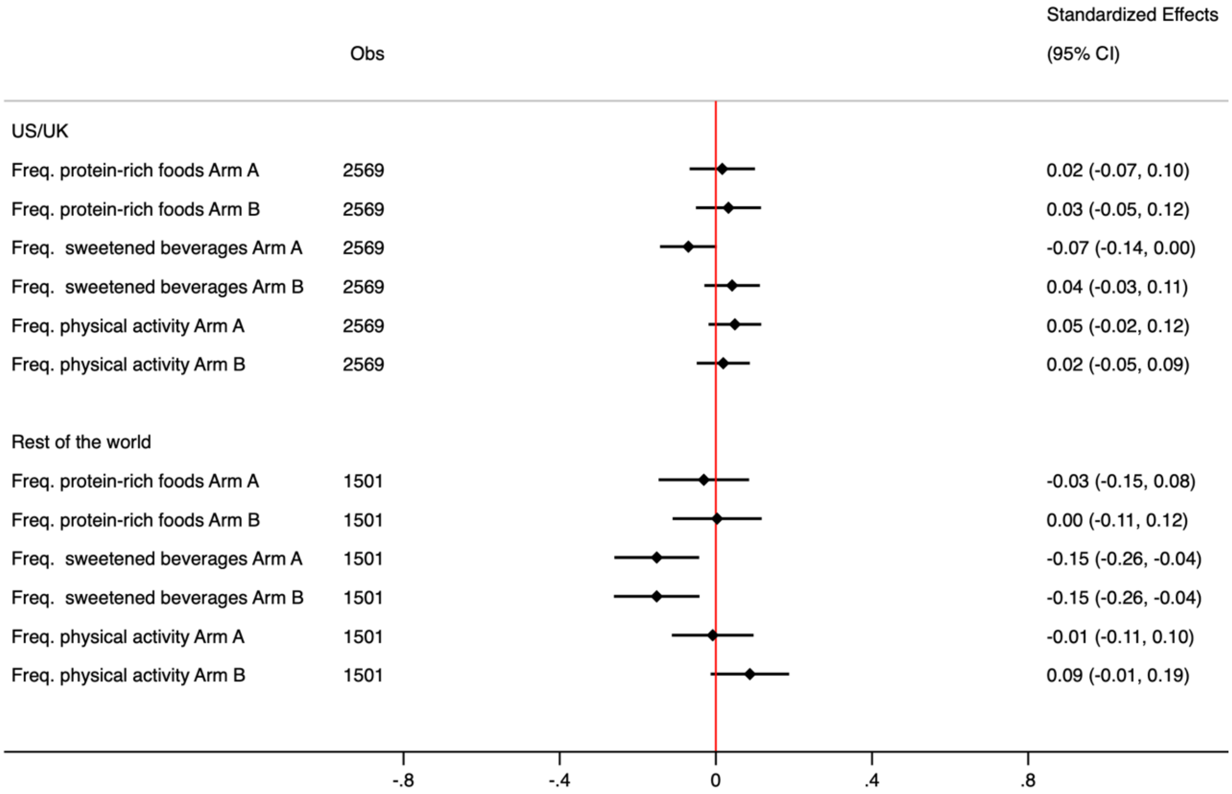

Note: Arm A refers to Written Microsteps + storytelling video booster and Arm B to Written Microsteps + didactic video booster.

© 2026 Adam M et al. *JAMA Network Open*.

eTable 1. Descriptive Statistics for Baseline Outcome Variables

**Variable, No. (%)**	**Total sample (n=5,054)**	Storytelling Arm A (n=1692)	Didactic Arm B (n=1,689)	Do-nothing Control (n=1673)
**Microsteps, No. (%)**				
Likely to drink caffeine after 2pm	2600 (51.4)	884 (52.2)	876 (51.9)	840 (50.2)
Likely to set a bedtime reminder	1491 (29.5)	549 (32.4)	475 (28.1)	467 (27.9)
Likely to swap sugary drinks for water	3881 (76.8)	1275 (75.4)	1316 (77.9)	1290 (77.1)
Likely to include protein at each meal	4578 (90.6)	1540 (91.0)	1526 (90.3)	1512 (90.4)
Likely to schedule movement time	2843 (56.3)	969 (57.3)	925 (54.8)	949 (56.7)
Likely to add movement everyday	3295 (65.2)	1117 (66.0)	1089 (64.5)	1089 (65.1)
Likely to breathe when stressed	3804 (75.3)	1287 (76.1)	1245 (73.7)	1272 (76.0)
Likely to go outside for 5 min without devices	3502 (69.3)	1181 (69.8)	1153 (68)	1168 (69.8)
**Hope & Happiness Thermometers (0-100), (SD)**				
Hope	71.8 (20.4)	72.2 (20.5)	71.5 (20.1)	71.7 (20.7)
Happiness	70.4 (22.1)	70.9 (21.9)	70.0 (21.8)	70.2 (22.7)
**Self-reported behaviors No. (%)**				
≥ several times a week protein-rich foods at each meal	4310 (85.3)	1453 (85.9)	1427 (84.5)	1430 (85.5)
≥ several times a week sweetened beverages	1736 (34.3)	578 (34.2)	578 (34.2)	580 (34.7)
≥ several times a week in physical activity	3189 (63.1)	1064 (62.9)	1072 (63.5)	1053 (62.9)

eTable 2. Multiple Testing *P* Value Adjustment for T1 Outcomes (Romano Wolf Method)

	**Model p-value**	**Resample p-value**	**-Wolf p-value**
**Hope**			
Arm A (storytelling)	0.0000	0.0010	0.0010
Arm B (didactic)	0.0000	0.0010	0.0010
**Happiness**			

© 2026 Adam M et al. *JAMA Network Open*.

Arm A (storytelling)	0.0000	0.0010	0.0010
Arm B (didactic)	0.0000	0.0010	0.0010
**No Caffeine after 2pm.**			
Arm A (storytelling)	0.0000	0.0010	0.0010
Arm B (didactic)	0.0000	0.0010	0.0010
**Set reminder to bed**			
Arm A (storytelling)	0.0000	0.0010	0.0010
Arm B (didactic)	0.0000	0.0010	0.0010
**Sugar drinks to water**			
Arm A (storytelling)	0.0000	0.0010	0.0010
Arm B (didactic)	0.0000	0.0010	0.0010
**Protein during meals**			
Arm A (storytelling)	0.0000	0.0010	0.0010
Arm B (didactic)	0.0000	0.0010	0.0010
**Schedule movement time**			
Arm A (storytelling)	0.0000	0.0010	0.0010
Arm B (didactic)	0.0000	0.0010	0.0010
**More movement everyday**			
Arm A (storytelling)	0.0000	0.0010	0.0010
Arm B (didactic)	0.0000	0.0010	0.0010
**Breathe when stressed**			
Arm A (storytelling)	0.0000	0.0010	0.0010
Arm B (didactic)	0.0000	0.0010	0.0010
**Go outside 5 minutes**			
Arm A (storytelling)	0.0000	0.0010	0.0010
Arm B (didactic)	0.0000	0.0010	0.0010

© 2026 Adam M et al. *JAMA Network Open*.

eTable 3. Multiple Testing *P* Value Adjustment for T2 Outcomes (Romano Wolf Method)

	Model p-value	Resample p-value	Romano Wolf p-value
**Hope**			
Arm A (storytelling)	0.8243	0.8322	0.9970
Arm B (didactic)	0.9511	0.9560	0.9970
**Happiness**			
Arm A (storytelling)	0.8556	0.8462	0.9970
Arm B (didactic)	0.5642	0.5774	0.9920
**No Caffeine after 2pm.**			
Arm A (storytelling)	0.0000	0.0010	0.0010
Arm B (didactic)	0.0000	0.0010	0.0010
**Set reminder to bed**			
Arm A (storytelling)	0.0000	0.0010	0.0010
Arm B (didactic)	0.0000	0.0010	0.0010
**Sugar drinks to water**			
Arm A (storytelling)	0.0003	0.0010	0.0050
Arm B (didactic)	0.0031	0.0050	0.0310
**Freq. protein-rich foods**			
Arm A (storytelling)	0.0004	0.0020	0.0070
Arm B (didactic)	0.0001	0.0010	0.0040
**Schedule movement time**			
Arm A (storytelling)	0.0000	0.0010	0.0010
Arm B (didactic)	0.0000	0.0010	0.0010
**More movement everyday**			
Arm A (storytelling)	0.0000	0.0010	0.0040
Arm B (didactic)	0.0001	0.0020	0.0040
**Breathe when stressed**			
Arm A (storytelling)	0.0001	0.0010	0.0040
Arm B (didactic)	0.0005	0.0010	0.0070

© 2026 Adam M et al. *JAMA Network Open*.

**Go outside 5 minutes**			
Arm A (storytelling)	0.0000	0.0010	0.0010
Arm B (didactic)	0.0012	0.0020	0.0130
**Freq. sweetened beverages**			
Arm A (storytelling)	0.0021	0.0020	0.0230
Arm B (didactic)	0.5561	0.5774	0.9920
**Freq. protein-rich foods**			
Arm A (storytelling)	0.9267	0.9351	0.9970
Arm B (didactic)	0.5392	0.5475	0.9920
**Freq. physical activity**			
Arm A (storytelling)	0.3489	0.3477	0.9540
Arm B (didactic)	0.1533	0.1528	0.7113

© 2026 Adam M et al. *JAMA Network Open*.
