# Supplementary material for: Digital Microsteps as Scalable Adjuncts for Adults Using GLP-1 Receptor Agonists: A Randomized Clinical Trial
Source: JAMA Netw Open. 2026 Mar 9;9(3):e260577. doi: 10.1001/jamanetworkopen.2026.0577 (PMC12973101; doi:10.1001/jamanetworkopen.2026.0577)
Supplement: Supplement 2. — Trial Protocol [file jamanetwopen-e260577-s002.pdf]

PROTOCOL APPLICATION FORM
Human Subjects Research
Stanford University

Title : Short, animated storytelling video and written messages to promote adoption of health "microsteps": an online randomized experiment

Approval Period: 06/13/2025 - 12/31/2999

Personnel Info 2
Participant Population 2
Study Location 3
General Checklist 3
Funding 4
Resources 5
Exempt Paragraph(s) 6
Purpose, Study Procedures 9
Participant Population(a-e) 11
Participant Population(f-i) 12
Risks 12
Privacy And Confidentiality 13
Conflict Of Interest 14
Consent Background 15
Assent Background 16
Attachments 16
Obligations 16

Title : Short, animated storytelling video and written messages to promote adoption of health "microsteps": an online randomized experiment
Approval Period: 06/13/2025 - 12/31/2999

Protocol Director

Name		Degree (Program/year if student)		Position, e.g. Assistant Professor, Resident, etc.
Maya Adam		MD, PhD		Clinical Associate Professor
**Department**		**Phone**		**E-mail**
Pediatrics - Infectious Diseases		(650) 839-3600		madam@stanford.edu
**CITI Training current**				Y

Admin Contact

Name		Degree (Program/year if student)		Position, e.g. Assistant Professor, Resident, etc.
Maya Adam		MD, PhD		Clinical Associate Professor
**Department**		**Phone**		**E-mail**
Pediatrics - Infectious Diseases		(650) 839-3600		madam@stanford.edu
**CITI Training current**				Y

Investigator

Name		Degree (Program/year if student)		Position, e.g. Assistant Professor, Resident, etc.
Eleni Linos		MD, PhD		Professor
**Department**		**Phone**		**E-mail**
Dermatology		6508393600		madam@stanford.edu
**CITI Training current**				Y

Other Contact

Name		Degree (Program/year if student)		Position, e.g. Assistant Professor, Resident, etc.
**Department**		**Phone**		**E-mail**
**CITI Training current**				

Academic Sponsor

Name		Degree (Program/year if student)		Position, e.g. Assistant Professor, Resident, etc.
**Department**		**Phone**		**E-mail**

Other Personnel

Participant Population(s) Checklist — Yes/No

- Children (under 18) — N
- Pregnant Women and Fetuses — N

- Neonates (0 - 28 days) N
- Abortuses N
- Prisoners N
- International Participants N
  Please enter the countries separated by comma
- Impaired Decision Making Capacity N
- Cancer Subjects N
- Laboratory Personnel N
- Healthy Volunteers N
- Students N
  Stanford students N Other students N
- Employees N
- Other (i.e., any population that is not specified above) Y

Study Location(s) Checklist — Yes/No

- Stanford University Y
- Clinical & Translational Research Unit (CTRU) N
- Stanford Medicine Health Care Y
  Tri-Valley N
- Stanford Medicine Children's Health N
- VAPAHCS (Specify PI at VA)
- Other (Specify other study locations)

General Checklist

Multi-site — Yes/No

- Is this a multi-site study? A multi-site study uses the same protocol to conduct human subjects research at more than one site. N

Cooperative/Collaborative Study? — Yes/No

- Are there any collaborating institution(s)? A collaborating institution is generally an institution that collaborates equally on a research endeavor with one or more institutions. Y

Institution Name	Contact Name	Contact Phone	Contact Email	Permission?	Engaged?
Department of Psychiatry at the Vagelos College of	Doron Amsalem		doron.amsalem@nyspi.columbia.edu	Y	N

Cancer Institute	Yes/No
• Cancer-Related Studies (studies with cancer endpoints), Cancer Subjects (e.g., clinical trials, behavior/prevention) or Cancer Specimens (e.g., blood, tissue, cells, body fluids with a scientific hypothesis stated in the protocol).	N

Tissues and Specimens	Yes/No
• Human blood, cells, tissues, or body fluids (tissues)?	N
• Tissues to be stored for future research projects?	N
• Tissues to be sent out of this institution as part of a research agreement? For guidelines, please see Material Transfer Agreements	N
• Human Embryos or Gametes?	N

Veterans Affairs (VA)	Yes/No
• The research recruits participants at the Veterans Affairs Palo Alto Health Care System(VAPAHCS).	N
• The research involves the use of VAPAHCS non-public information to identify or contact human research participants or prospective subjects or to use such data for research purposes.	N
• The research is sponsored (i.e., funded) by VAPAHCS.	N
• The research is conducted by or under the direction of any employee or agent of VAPAHCS (full-time, part-time, intermittent, consultant, without compensation (WOC), on-station fee-basis, on-station contract, or on-station sharing agreement basis) in connection with her/his VAPAHCS responsibilities.	N
• The research is conducted using any property or facility of VAPAHCS.	N

Payment	Yes/No
• Subjects will be paid/reimbursed for participation? See payment considerations.	Y

Funding	Yes/No
• Training Grant?	N
• Program Project Grant?	N
• Federally Sponsored Project?	N

Funding

Funding - Grants/Contracts/Agreements

Title : Short, animated storytelling video and written messages to promote adoption of health "microsteps": an online randomized experiment
Approval Period: 06/13/2025 - 12/31/2999

Funding - Fellowships

Gift Funding

Dept. Funding

Other Funding

Other Fund Name : Adam Digital Health Fund

Other Fund Name : Department of Psychiatry at the Vagelos College of Physicians and Surgeons of Co

Resources :

a) **Qualified staff.**

Please state and justify the number and qualifications of your study staff.

Because this is an online study, conducted on academic research platform, no study staff are required. The co-investigators involved in designing the study and the intervention as well as analyzing the data are medical doctors and faculty at either Stanford or the Department of Psychiatry at the Vagelos College of Physicians and Surgeons of Columbia University.

b) **Training.**

Describe the training you will provide to ensure that all persons assisting with the research are informed about the protocol and their research-related duties and functions.

Because this is an online study, conducted on academic research platform, no study staff are required. The co-investigators involved in designing the study and the intervention as well as analyzing the data are medical doctors and faculty at either Stanford or the Department of Psychiatry at the Vagelos College of Physicians and Surgeons of Columbia University.

c) **Facilities.**

Provide the location(s) where the research will be conducted, including physical address if not conducted on site at Stanford University, Stanford Hospital on Pasteur Dr., Lucile Packard Children's Hospital on Welch Rd. or VAPAHCS. Describe the facilities and resources available to conduct the research at these sites.

No facilities required.

d) **Sufficient time.**

Explain the time that you and your research team will allocate to perform the research activities, including data analysis.

Questionnaire surveys will be collected online using the Prolific and Qualtrics platforms within 120 days.

e) **Access to target population.**

Explain and justify whether you will have access to a population that will allow recruitment of the required number of participants.

We will recruit participants from the recruitment platform Prolific. Prolific specializes in recruiting the required number of participants for the study. Participants will be eligible if they are between the ages of 18-50, the age range of adults who are most likely to be users of social media and therefore reflect our target population.

f) Access to resources if needed as a consequence of the research.

State whether you have medical or psychological resources available that participants might require as a consequence of the research when applicable. Please describe these resources.

The online participants will be anonymous to the Principal Investigator.

g) Lead Investigator or Coordinating Institution in Multi-site Study.

Please explain (i) your role in coordinating the studies, (ii) procedures for routine communication with other sites, (iii) documentation of routine communications with other sites, (iv) planned management of communication of adverse outcomes, unexpected problems involving risk to participants or others, protocol modifications or interim findings.

Title

Short, animated storytelling video and written messages to promote adoption of health "microsteps": an online randomized experiment

Exempt Form

In order to qualify as Exempt, a protocol must be no more than minimal risk AND must only involve human subjects in one or more of the following paragraphs.

Select one or more of the following paragraphs:

1. N **Research, conducted in established or commonly accepted educational settings, that specifically involves normal educational practices that are not likely to adversely impact students opportunity to learn required educational content or the assessment of educators who provide instruction. This includes most research on regular and special education instructional strategies, and research on the effectiveness of or the comparison among instructional techniques, curricula, or classroom management methods.**

2. N **Research that only includes interactions involving educational tests (cognitive, diagnostic, aptitude, achievement), adult surveys, and interviews, (includes group interviews/focus groups under 2ii only), or observation of public behavior (including visual or auditory recording) if at least one of the following criteria is met:**

i) N The information obtained is recorded by the investigator in such a manner that the identity of the human subjects cannot readily be ascertained, directly or through identifiers linked to the subjects;

ii) N Any disclosure of the human subjects responses outside the research

would not reasonably place the subjects at risk of criminal or civil liability or be damaging to the subjects financial standing, employability, educational advancement, or reputation; or

iii) N The information obtained is recorded by the investigator in such a manner that the identity of the human subjects can readily be ascertained, directly or through identifiers linked to the subjects, and an IRB conducts a limited IRB review to make the determination required by ? .111(a)(7)

3 Y **(i)Research involving benign behavioral interventions in conjunction with the collection of information from an adult subject through verbal or written responses (including data entry) or audiovisual recording if the subject prospectively agrees to the intervention and information collection and at least one of the following criteria is met: See (3)(ii) below for more on the definition of a benign behavioral intervention.**

A) Y The information obtained is recorded by the investigator in such a manner that the identity of the human subjects cannot readily be ascertained, directly or through identifiers linked to the subjects;

B) N Any disclosure of the human subjects responses outside the research would not reasonably place the subjects at risk of criminal or civil liability or be damaging to the subjects financial standing, employability, educational advancement, or reputation; or

C) N The information obtained is recorded by the investigator in such a manner that the identity of the human subjects can readily be ascertained, directly or through identifiers linked to the subjects, and an IRB conducts a limited IRB review to make the determination required by ? .111(a)(7).

ii) **For the purpose of this provision, benign behavioral interventions are brief in duration, harmless, painless, not physically invasive, not likely to have a significant adverse lasting impact on the subjects, and the investigator has no reason to think the subjects will find the interventions offensive or embarrassing. Provided all such criteria are met, examples of such benign behavioral interventions would include having the subjects play an online game, having them solve puzzles under various noise conditions, or having them decide how to allocate a nominal amount of received cash between themselves and someone else.**

iii) **If the research involves deceiving the subjects regarding the nature or purposes of the research, this exemption is not applicable unless the subject authorizes the deception through a prospective agreement to participate in research in circumstances in which the subject is informed that he or she will be unaware of or misled regarding the nature or purposes of the research.**

N **Is deception involved?**

4. N **Secondary research for which consent is not required: Secondary research uses of identifiable private information or identifiable biospecimens, if at least one of the following criteria is met:**

i) N The identifiable private information or identifiable biospecimens are publicly available;

ii) N Information, which may include information about biospecimens, is recorded by the investigator in such a manner that the identity of the human subjects cannot readily be ascertained directly or through identifiers linked to the subjects, the investigator does not contact the subjects, and the investigator will not re-identify subjects;

iii) The research involves only information collection and analysis involving the investigator's use of identifiable health information when that use is regulated under 45 CFR parts 160 and 164, subparts A and E, for the purposes of "health care operations" or "research" as those terms are defined at 45 CFR 164.501 or for "public health activities and purposes" as described under 45 CFR 164.512(b); or

iv) N The research is conducted by, or on behalf of, a Federal department or agency using government-generated or government-collected information obtained for nonresearch activities, if the research generates identifiable private information that is or will be maintained on information technology that is subject to and in compliance with section 208(b) of the E-Government Act of 2002, 44 U.S.C. 3501 note, if all of the identifiable private information collected, used, or generated as part of the activity will be maintained in systems of records subject to the Privacy Act of 1974, 5 U.S.C. 552a, and, if applicable, the information used in the research was collected subject to the Paperwork Reduction Act of 1995, 44 U.S.C. 3501 et seq.

5. N **Research and demonstration projects that are conducted or supported by a Federal department or agency, or otherwise subject to the approval of department or agency heads (or the approval of the heads of bureaus or other subordinate agencies that have been delegated authority to conduct the research and demonstration projects), and that are designed to study, evaluate, improve, or otherwise examine public benefit or service programs, including procedures for obtaining benefits or services under those programs, possible changes in or alternatives to those programs or procedures, or possible changes in methods or levels of payment for benefits or services under those programs. Such projects include, but are not limited to, internal studies by Federal employees, and studies under contracts or consulting arrangements, cooperative agreements, or grants. Exempt projects also include waivers of otherwise mandatory requirements using authorities such as sections 1115 and 1115A of the Social Security Act, as amended.**

i) Each Federal department or agency conducting or supporting the research and (i) demonstration projects must establish, on a publicly accessible Federal Web site or in such other manner as the department or agency head may determine, a list of the research and demonstration projects that the Federal department or agency conducts or supports under this provision. The research or demonstration project must be published on this list prior to commencing the research involving human subjects.

ii) [Reserved]

6. N **Taste and food quality evaluation and consumer acceptance studies:**

i) If wholesome foods without additives are consumed, or

ii) N If a food is consumed that contains a food ingredient at or below the level and for a use found to be safe, or agricultural chemical or environmental contaminant at or below the level found to be safe, by the Food and Drug Administration or approved by the Environmental Protection Agency or the Food Safety and Inspection Service of the U.S. Department of Agriculture.

7. **Reserved for future use.**

8. **Reserved for future use.**

Title : Short, animated storytelling video and written messages to promote adoption of health "microsteps": an online randomized experiment
Approval Period: 06/13/2025 - 12/31/2999

1. Purpose

a) **In layperson's language state the purpose of the study in 3-5 sentences.**

The purpose of the study is to compare the effectiveness of two different approaches for promoting the adoption of health "microsteps" - using written messages with a short instructional video or written messages with a short, animated story video - in 6000 online adults who have experience taking GLP-1 agonists (medications like Ozemoic and Wegovy).
We will measure differences in health microstep adoption post intervention and two weeks later (T=0 and T=14 days later) between the two intervention arms and a do-nothing control arm.
Intervention A arm will read the written health messages and watch the instructional video. Intervention B arm read the written messages and watch the story video, and the do-nothing control arm will only complete the surveys.
All three groups will be offered post-trial access to the video content and the written health messages at the end of the trial (after the 14-day follow up).
The videos can be viewed here:
https://youtu.be/tE32V5KSlc0?si=CLMOfTebuPk-rcEP
https://drive.google.com/file/d/1JiUNJnkUA-d1I4bcnGho-22g-iv8TQMl/view?usp=drive_link

b) **State what the Investigator(s) hope to learn from the study. Include an assessment of the importance of this new knowledge.**

Research has highlighted the critical role of health behavior changes to augment health outcomes in people taking GLP-1 agonists. While this medication has proven effective for improving blood sugar control and weight management, it is even more effective when combined with behavior change. Yet, motivating behavior change in people who struggle with weight management and/or blood sugar control can be challenging. To address this challenge, we will test two simple "microstep" messaging approaches. Microsteps are small behavior changes developed as part of Arianna Huffington's Thrive Global public health program. Additionally, we have designed a short, animated story video to underscore these behavioral recommendations. Short, animated storytelling has been previously shown to enhance engagement of the public in health recommendations. The video can be rapidly distributed through social media channels, overcoming barriers to access. To evaluate if written messages describing behavioral microsteps are adopted among GLP-1 agonist users, and to test if short, animated storytelling videos, vs. instructional videos, can augment such adoption, we propose to randomize 6000 adult GLP-1 agonist users to receive either the written messages and an instructional video or the written messages with a story video. Intention to adopt and self-reported actual adoption of the microsteps will be compared between these two groups and both groups will be compared with a do-nothing control group.

c) **Explain why human subjects must be used for this project.** (**i.e. purpose of study is to test efficacy of investigational device in individuals with specific condition; purpose of study is to examine specific behavioral traits in humans in classroom or other environment**)

Human subjects are needed because the purpose of this study is to test whether written messages and instructional video content or written messages supported by short, animated storytelling video content, can boost adoption of health behaviors in human subjects who struggle with weight management or blood sugar control, and are therefore taking a GLP-1 agonist. Because the US and the UK are countries where many people struggle with weight management and blood sugar control, we have chosen to recruit participants from these two countries.

2. Study Procedures

a) i) **Describe ALL the procedures human participants will undergo. ii)Are the research procedures the least risky that can be performed consistent with sound research design? iii)For research involving collaborators, please specify the respective roles of Stanford and each collaborator on the protocol.**

This will be an online study setting. We will use the academic recruitment platforms Prolific Academic (ProA: https://www.prolific.co/) to recruit the study participants. When these participants sign up on Prolific

to take part in various studies, they fill out Prolific's demographic data questionnaires. If Prolific users wish to volunteer that they are taking certain common medications, they can do so. One of the optional questions they can answer is "Are you currently taking a GLP-1 agonist (a medication like Ozemic or Wegovy)?" When we are recruiting participants for our study, we can screen for participants who answered "yes" to that question. We can also screen and add participants who answer "yes" to the question: "Have you taken a GLP-1 agonist in the past?" All participants remain anonymous to the research team at all times and we will not solicit any information from them that could be used to identify them. We also will not solicit any protected health information from the participants.

The ProA platform includes participants from the United States and the United Kingdom, with more that 9000 users who have volunteered that they are either currently taking a GLP-1 agonist or did so in the past. Participants will see their intervention and complete their surveys on an online web survey platform called Stanford Qualtrics. Qualtrics is an experiment builder that provides users with the tools for undertaking online behavioral research.

Intervention A arm will read the written health messages then watch the instructional video. Intervention B arm will read the written messages then watch the story video, and the do-nothing control arm will only complete the surveys. Two weeks later, all participants will be invited back to complete follow-up surveys. All three groups will be offered post-trial access to the video content and the written health messages at the end of the trial (after the 14-day follow up).
The videos can be viewed here:
https://youtu.be/tE32V5KSlc0?si=CLMOfTebuPk-rcEP
https://drive.google.com/file/d/1JiUNJnkUA-d1I4bcnGho-22g-iv8TQMl/view?usp=drive_link

Prolific participants will read an Information Sheet, delivered to them on the Prolific platform, describing the purpose of the study, as well as any potential risks (minimal to none) and benefits (like learning about healthy behaviors). They will also be reminded of their rights as a study participant and, after reviewing that document, they can decide if they consent to take part in the study or not.

All research procedures involve very minimal or no risk to the participants and they are consistent with sound research design.

Stanford faculty will lead intervention design, implementation and writing/disseminating of the study findings.

b) Explain how the above research procedures are the least risky that can be performed consistent with sound research design.

There are no foreseeable risks to participating in this online study.
Participants volunteer and consent to participate in the study and can withdraw at any time.

c) State if deception will be used. If so, provide the rationale and describe debriefing procedures. Since you will not be fully informing the participant in your consent process and form, complete an alteration of consent (in section 9). Submit a debriefing script (in section 11).

No deception will be used.

d) State if photo, audio or video recording will occur. Describe what will become of the photos or recording after use, e.g., shown at scientific meetings, erased. Describe the final disposition of the recordings.

Yes, the treatment or intervention is a written description of health "microsteps", an instructional video, and an educational-entertainment
video about healthy screen use, getting enough sleep, going for walks in the fresh air if possible and drinking enough water. The videos can be viewed here:
https://youtu.be/tE32V5KSlc0?si=CLMOfTebuPk-rcEP and here:
https://drive.google.com/file/d/1JiUNJnkUA-d1I4bcnGho-22g-iv8TQMl/view?usp=drive_link

Title : Short, animated storytelling video and written messages to promote adoption of health "microsteps": an online randomized experiment
Approval Period: 06/13/2025 - 12/31/2999

3. Background

a) Describe past findings leading to the formulation of the study.

A substantial body of research supports the need to incorporate behavior changes to augment medicines aimed at helping people manage their weight and blood sugar. Our prior studies have suggested that short, animated storytelling videos have the capacity to spread health information broadly to the public and impact attitudes. Other research suggests that instructional videos may also support behavior change. Behavioral "microsteps" have been proven effective because they are small changes in the right direction, that can add up to significant health benefits.

4. Participant Population

a) State the following: (i) the number of participants expected to be enrolled at Stanford-affiliated site(s); (ii)the total number of participants expected to enroll at all sites; (iii) the type of participants (i.e. students, patients with certain cancer, patients with certain cardiac condition) and the reasons for using such participants.

This will be an online study setting. The academic recruitment platform Prolific Academic (ProA: https://www.prolific.co/) will be used to recruit the study participants. The ProA platform is global in scope and includes study participants from the US and the UK.

i) It is unknown if participants will be recruited from a Stanford affiliated site. ProA recruits participants at a national level. ii) ProA will recruit 6000 participants. iii) ProA will recruit adult men and women aged 18-99 years who have reported that they have experience taking a GLP-1 agonist. This age group is selected because they are adults who may have experience using a GLP-1 agonist to manage their weight or blood sugar.

b) State the age range, gender, and ethnic background of the participant population being recruited.

The age range will be 18-99 years. ProA will recruit participants from its participant pool. No specific gender or ethnic background is being recruited.

c) State the number and rationale for involvement of potentially vulnerable subjects in the study (including children, pregnant women, economically and educationally disadvantaged, decisionally impaired, homeless people, employees and students). Specify the measures being taken to minimize the risks and the chance of harm to the potentially vulnerable subjects and the additional safeguards that have been included in the protocol to protect their rights and welfare.

This is an online study. All participants will be anonymous to the research team. ProA will handle the recruitment. Participants will watch an animated video, or an instructional video, and read written messages about health "microsteps". There is little or no chance of study participants being harmed.

d) Describe your plan to identify and recruit potential participants including who will inform them about the study and how they will be initially contacted by the researchers (e.g., Participant Engagement services; chart review; treating physician; ads including social media posts). All final or revised recruitment materials must be approved by the IRB before use. Contacting potential participants is not permitted prior to IRB approval. See Recruitment Guidance for additional information.

ProA will handle the recruitment.
These platform users choose to be recruited by the platforms. To be chosen, the user must sign-up by visiting their websites at https://app.prolific.co/register/participant. To register, users must agree to the terms and conditions and the privacy policy. These documents can be found at:
https://www.prolific.co/assets/docs/Participant_Terms.pdf
https://www.prolific.co/assets/docs/Prolific_privacy-policy.pdf

Please see this open-access, peer-reviewed publication explaining how Prolific Academic handles recruitment of participants:

https://www.sciencedirect.com/science/article/pii/S2214635017300989

The study investigators do NOT have access to any contact information of individual personal data from the participants.

The consent form itself is the only language that the survey platform will use when advertising or recruiting this study to potential participants.

Because the participants in this fully online trial are all subscribed to Prolific Academic as potential study participants, we do not need separate brochures or other recruitment materials. (Prolific recruits volunteers from their subscriber base.)

e) Inclusion and Exclusion Criteria.

Identify inclusion criteria.

All adult participants who answered "yes" to the question about whether they have experience taking a GLP-1 agonist, and are between the ages of 18 and 99 years, that are registered on the ProA platform, will be eligible to participate in the study. Participants must have reading competency in English to participate in the study.

Identify exclusion criteria.

Exclusion criteria for all data collection is those who do not meet the above listed inclusion criteria. If a participant reports an out of range age or cannot read English, the ProA platform will inform the participant that they are not eligible for participation in our study.

f) Describe your screening procedures, including how qualifying laboratory values will be obtained. If you are collecting personal health information prior to enrollment (e.g., telephone screening), please request a limited waiver of authorization in section #10.

ProA will handle the screening procedures. More information can be found here:
https://researcher-help.prolific.co/hc/en-gb/articles/360009221093-Using-our-demographic-filters-to-prescreen-participants

g) Payment/reimbursement. Explain the amount and schedule of payment or reimbursement, if any, that will be paid for participation in the study. Substantiate that proposed payments are reasonable and commensurate with the expected contributions of participants and that they do not constitute undue pressure on participants to volunteer for the research study. Include provisions for prorating payment. See payment considerations

Study participants will be compensated $4.80 for completing the survey at ProA. Participants will be paid $2.40 after completing the first 12-min "step" of the trial, and then another $2.40 after completing the second "step" of the study (in which they are asked to complete the short survey a second time after 14 days have passed). ProA will make all reward payments to study participants. As per Prolific guidance, $12/hr is considered "good" payment and is above minimum wage. Our participants will spend no more than 12 minutes in the first step of the trial (based on previous studies we have conducted) and less than 12 minutes in the second step, since they are only being asked to complete the very short questionnaire in this follow-up step.

h) Costs. Please explain any costs that will be charged to the participant.

Zero costs will be charged to the participant.

i) Estimate the probable duration of the entire study. Also estimate the total time per participant for: (i) screening of participant; (ii) active participation in study; (iii) analysis of participant data.

The total time of the study will be 3 months. i) ProA will undertake the screening process, and is therefore unknown to the PI. ii) The first step of the study will take 12 minutes. iii) After 14 days, participants will be asked to complete the short survey again. This will take less time than the first step (less that 12 minutes) and iv) One month to analyze the data.

5. Risks

a) Describe risks. Include risks to privacy, confidentiality, etc..

There are no foreseeable risks involved in participating in this study.

b) If you are conducting international research, describe the qualifications/preparations that enable you to both estimate and minimize risks to participants. Provide an explanation as to why the research must be completed at this location and complete the International Research Form. If not applicable, enter N/A.

This study will compare the effect of written messages aimed at promoting health behavior changes, to written messages plus an animated video aimed at promoting health behaviors, to a control condition. By using a trusted academic research platform, we are minimizing the risks to participants through this platform's stringent participant privacy, protection and data security protocols.

c) Could any disclosure of the participant's response outside the research reasonably place them at risk of loss of insurability, criminal or civil liability, or be damaging to the participant's financial standing, employability, or reputation?

No.

6. Benefits

a) Describe the potential benefit(s) to be gained by the participants or by the acquisition of important knowledge which may benefit future participants, etc.

Benefits may include a gaining an understanding about the people who suffer from addiction.

7. Privacy and Confidentiality

Privacy Protections

a) Describe the setting and method (e.g. crowded waiting room, patient exam room, telephone or email communication) in which interactions will occur and how the privacy interests of participants will be maintained. Note, high risk data such as PHI must be sent via "Secure:" email per Stanford policy.

Participation in this study will be entirely voluntary. The video, written messages and questionnaire survey will take approximately 12 minutes to complete on a single sitting. Upon follow-up, participants will only be asked to complete the short survey. This is expected to take less than 12 minutes (likely 10 min). Participants may withdraw their consent to participate in the study at any time before or during the study. However, participants will not be compensated for an incomplete survey. We will not use any data from an incomplete survey, except at a meta level where we report the aggregate number of incomplete surveys. Participants can only complete the survey once during each "step" of the study (ie, at T=0 and T=14 days later).

Participants must preview an information and consent form (see Appendix A) before they can begin the survey. The information and consent form describes the aims of the study, including possible risks and benefits. Participants will also be given a link to ProA's data privacy policy, which they initially agreed to when registering. We will provide participants with the contact details of the PI (MA) and the Stanford University Ethics Committee. We will inform participants that if they email the PI then their names could be revealed to us. The study investigators will keep this information

confidential. Consent to participate will be documented by clicking on a consent box on the consent form.

Because of the anonymized participant IDs, the study investigators can never meet or know the identity of the study participants. The study investigators cannot retrieve any personal information beyond what the study participants provide in the online survey. We will not collect any confidential information from participants, including their names. Data may only be passed to collaborating researchers in anonymized form. Third parties will not have access to the data. We do not consider ProA or Qualtrics to be a third party.

Confidentiality Protections

b) **Specify the PHI (protected health information) or other individually identifiable data or specimens you will obtain, use or disclose to others. PHI is health information linked to one or more of the HIPAA identifiers listed above. List BOTH health information AND identifiers.**

No PHI is collected. Additionally, since Qualtrics defaults to collecting participant IP addresses (which are identifiable information) but we plan to collect only anonymous data, we will enable Anonymize Responses in the Survey Options. Please see https://www.qualtrics.com/support/survey-platform/edit-survey/survey-options/survey-termination/#AnonymizingResponses for further information.

c) **Describe: (i) how data will be maintained (e.g., paper or electronic spreadsheet, desktop computer, laptop or other portable device); (ii) how you will maintain the confidentiality and data security, (e.g., password protected computer, encrypted files, locked cabinet and office); and (iii) who will have access to the data (e.g., research team, sponsors, consultants)**

All data collected in this study will be 100% de-identified, containing absolutely no PHI. Participants remain completely anonymous to all members of the research team at all times, as these participants are registered users of Prolific Academic and we will only see limited demographic data and survey responses that are collected via a Stanford Qualtrics survey. De-identified data will be downloaded from Qualtrics by the study team and data will be stored in an electronic spreadsheet on a password protected computer in a locked office.

d) **If sharing data with others, describe how data will be transferred or transmitted (e.g., file transfer software, file sharing, email). If transmitted via electronic networks, confirm a Stanford University IT approved platform will be used (see https://uit.stanford.edu/guide/riskclassifications) or that data will be encrypted while in transit. Additionally, confirm appropriate agreements are in place to allow for the sharing (see https://ico.stanford.edu/stanford-researchers/who-will-handle-my-agreement). If using or sharing PHI, refer to the following policies: https://uit.stanford.edu/security/hipaa.**

Only de-identified data (anonymous survey responses and limited demographic data containing NO PHI) will be downloaded from the Qualtrics survey platform directly to Dr. Adam's password-protected computer. We have previously consulted with ICO and no institutional agreement is necessary.

e) **If you plan to code the data, describe the method in which it will be coded and indicate who will have access to the key to the code.**

N/A

f) **How will you educate research staff to ensure they take appropriate measures to protect the privacy of participants and the confidentiality of data or specimens collected (e.g. conscious of oral and written communications, conducting insurance billing, and maintaining paper and electronic data)?**

All data collected will be anonymous.

8. Potential Conflict of Interest

Title : Short, animated storytelling video and written messages to promote adoption of health "microsteps": an online randomized experiment
Approval Period: 06/13/2025 - 12/31/2999

Investigators are required to disclose any outside interests that reasonably appear to be related/li to this protocol.

Outside Interest Tasks

Investigators	Role	Potential COI?	Date Outside Interest Answered	Date OPACS Disclosure Submitted	COI Review Determination
Maya Adam	PD	N	03/20/2025		N/A
Eleni Linos	COP D	N	03/20/2025		N/A

9. Consent Background

If the study involves an interaction with participants, attach an information sheet with your study details by clicking the ADD button below.

9. 1 Consent **Revised Information Sheet (Previously approved)**

Sponsor's Consent Version Number: (if any) :

a) Describe the informed consent process. Include the following.

i) Who is obtaining consent? (The person obtaining consent must be knowledgeable about the study.)

ii) When and where will consent be obtained?

iii) How much time will be devoted to consent discussion?

iv) Will these periods provide sufficient opportunity for the participant to consider whether or not to participate and sign the written consent?

v) What steps are you taking to minimize the possibility of coercion and undue influence?

vi) If consent relates to children and if you have a reason for only one parent signing, provide that rationale for IRB consideration.

People who are already enrolled as potential study participants on Prolific Academic, will be invited by Prolific Academic to review the detailed Information sheet attached to this protocol. Prolific users who choose to take part in the study, and meet eligibility criteria, will then be asked to indicate on Prolific that they consent to take part, and they will thereafter be able to access the survey link on Stanford Medicine's Qualtrics instance. Respondents will have plenty of time to review the Information sheet and they are all adults who are well aware of their rights as study participants, because they often take part in online studies on Prolific.

b) What is the Procedure to assess understanding of the information contained in the consent? How will the information be provided to participants if they do not understand English or if they have a hearing impairment? See HRPP Chapter12.2 for guidance.

Prolific ensures that all of their enrolled members have the capacity to assess and understand the information contained in the consent forms they consider. All Prolific-users are screened upon signing up on the platform. Additionally, all participants in our study need to be fluent in reading and writing in English

and they are made aware of this in the Information Sheet.

c) **What steps are you taking to determine that potential participants have the capacity to participate in the decision-making process? If your study may enroll adults who are unable to consent, describe (i) how you will assess the capacity to consent, (ii) what provisions will be taken if the participant regains the capacity to consent,(iii) who will be used as a legally authorized representative, and (iv) what provisions will be made for the assent of the participant.**

Prolific ensures that all of their enrolled members have the capacity to assess and understand the information contained in the consent forms they consider. All Prolific-users are screened upon signing up on the platform. Additionally, all participants in our study need to be fluent in reading and writing in English and they are made aware of this in the Information Sheet.

10. Assent Background (less than 18 years of age)

11. Attachments

Attachment Name	Attached Date	Attached By	Submitted Date
Revised Microsteps	06/02/2025	madam	
Revised Baseline and T1 Survey	06/13/2025	madam	
Revised FollowUp Survey	06/13/2025	madam	

Obligations

The Protocol Director agrees to:

- Adhere to principles of sound scientific research designed to yield valid results
- Conduct the study according to the protocol approved by the IRB
- Be appropriately qualified to conduct the research and be trained in Human Research protection, ethical principles, regulations, policies and procedures
- Ensure all Stanford research personnel are adequately trained and supervised
- Ensure that the rights and welfare of participants are protected including privacy and confidentiality of data
- Ensure that, when de-identified materials are obtained for research purposes, no attempt will be made to re-identify them.
- Disclose to the appropriate entities any potential conflict of interest
- Apply relevant professional standards.
- Any change or modification in the research protocol must be submitted to and approved by the IRB prior to the implementation of such change, except when necessary to eliminate apparent immediate hazards to the participant.
- For studies with expiration dates, submit a Continuing Review prior to the end of the approval period. An IRB Continuing Review (Renewal) Notice to Renew Protocol is sent to the Protocol Director prior to the expiration date of the protocol.
- Report promptly any new information, complaints, possibly serious and/or continuing noncompliance, or unanticipated problems involving risks to participants or others.

All data, including signed consent forms when applicable, must be retained for a minimum of three years past the completion of the research. Additional requirements may be imposed by your funding agency, your department, or other entities (e.g. 6 years for studies conducted under HIPAA or VAPAHCS). (See also Research Policy Handbook Retention of and Access to Research Data)

Questionnaries and Interview Guide

Y **By checking this box, I verify that I, as the Protocol Director (PD) responsible for this research protocol, have read and agree to abide by the above obligations, or that I have been delegated authority by the PD to certify that the PD has read and agrees to abide by the above obligations.**

APPROVAL LETTER/NOTICE NOTE: List all items (verbatim) that you want to be included in your approval letter (e.g., Amendment date, Investigator's Brochure version, consent form(s) version(s), advertisement name, etc.) in the box below.

Amendment date,.
