# Supplementary material for: Digital Microsteps as Scalable Adjuncts for Adults Using GLP-1 Receptor Agonists: A Randomized Clinical Trial
Source: JAMA Netw Open. 2026 Mar 9;9(3):e260577. doi: 10.1001/jamanetworkopen.2026.0577 (PMC12973101; doi:10.1001/jamanetworkopen.2026.0577)
Supplement: Supplement 3. — Data Sharing Statement [file jamanetwopen-e260577-s003.pdf]

Data Sharing Statement

Adam. Digital Microsteps as Scalable Adjuncts for Adults Using GLP-1 Receptor Agonists. *JAMA Netw Open*. Published March 05, 2026. doi:10.1001/jamanetworkopen.2026.0577

Data
Additional Information: Registered on 06/13/2025, ClinicalTrials.gov (NCT06967337).
Data available: Yes
Data types: Deidentified participant data
How to access data: Data is available upon request from the corresponding author: madam@stanford.edu
When available: With publication

Supporting Documents
Document types: None

Additional Information
Who can access the data: Researchers whose proposed use of the data has been approved.
Types of analyses: Health research.
Mechanisms of data availability: Without investigator support, after approval of a proposal.
